# Supplementary material for: Inflammatory signaling differentially changes chromatin accessibility and gene expression of the PD- associated kinase LRRK2 between human and mice
Source: Mol Neurodegener. 2026 Mar 18;21:24. doi: 10.1186/s13024-026-00938-0 (PMC13112740; doi:10.1186/s13024-026-00938-0)
Supplement: Supplementary file 1 — Supplementary Material 1 [file 13024_2026_938_MOESM1_ESM.zip › Supplementary/Supplementary Uncropped immunoblots.pdf]

Figure 1b

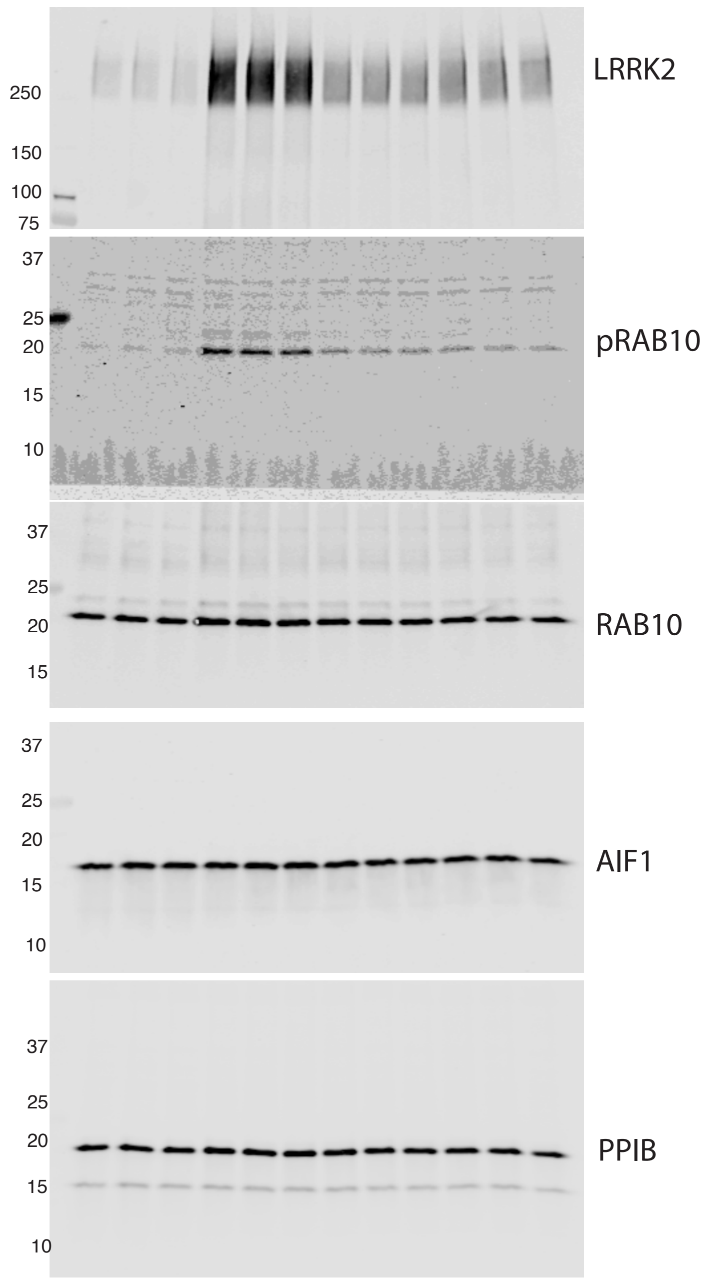

Figure 1e

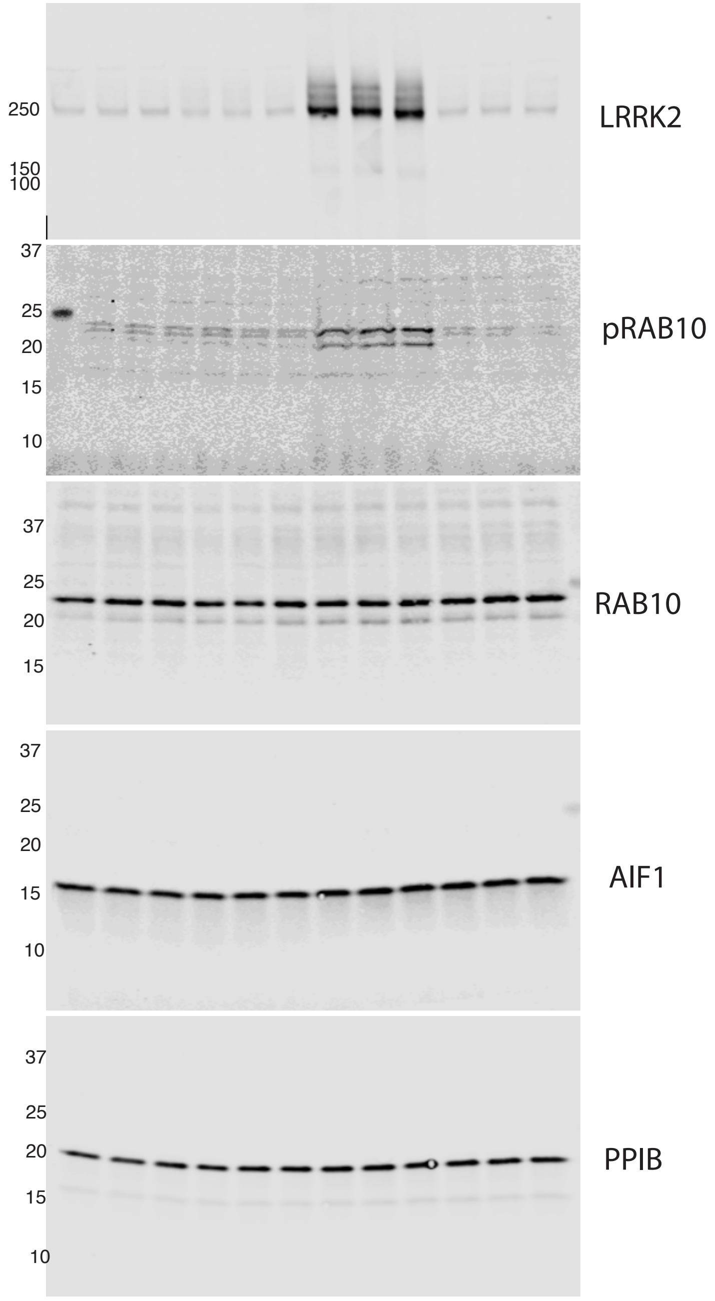

Figure 1b and e

Figure 1h

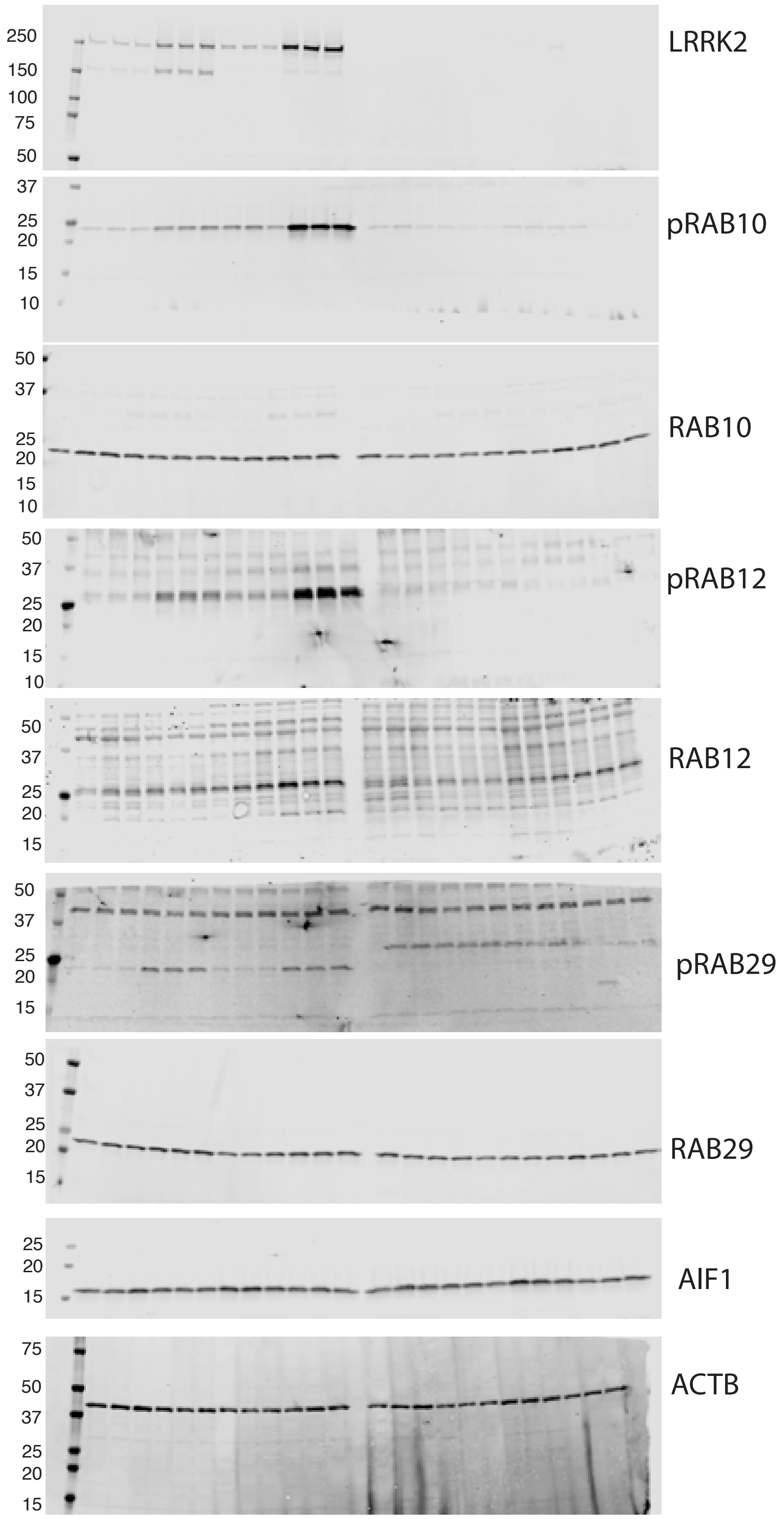

Figure 1h

Figure 2e

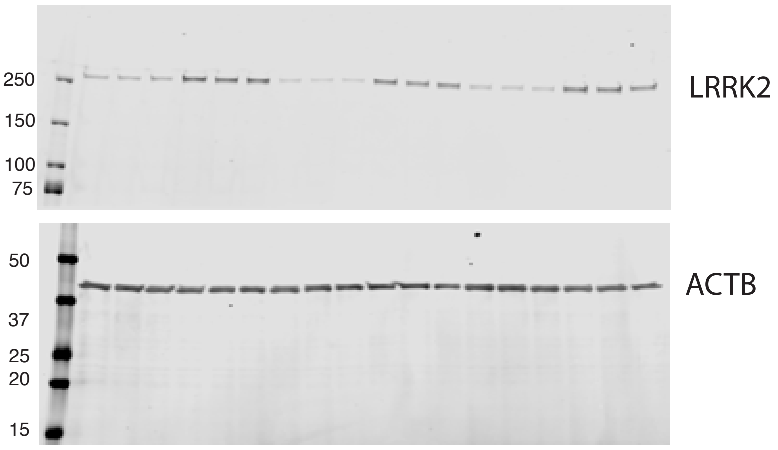

Figure 3e

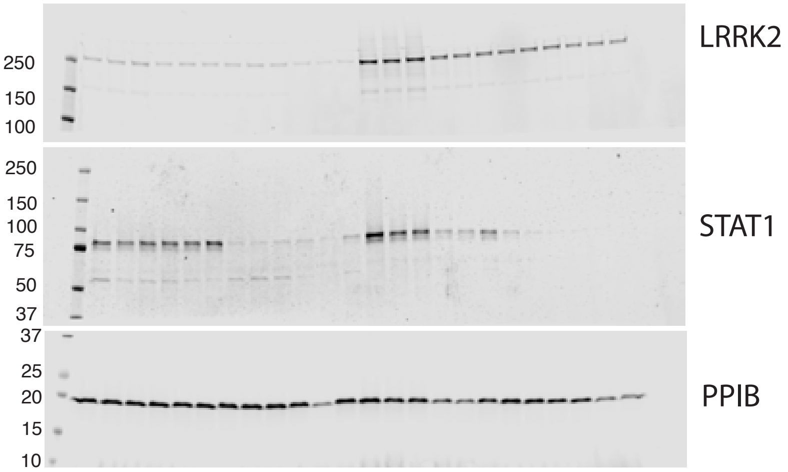

Figure 4e

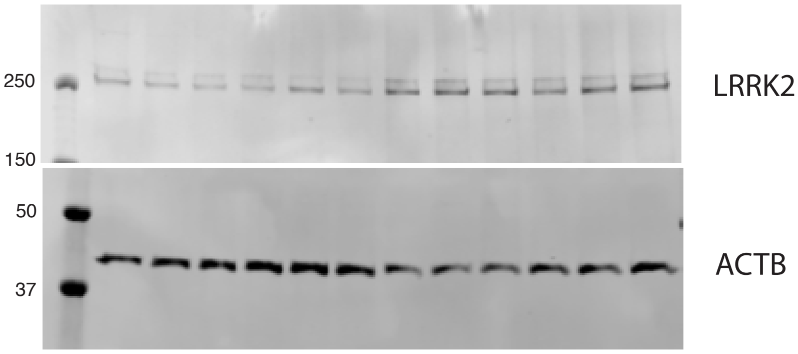

Figure 2e, 3e, 4e

Figure 6b

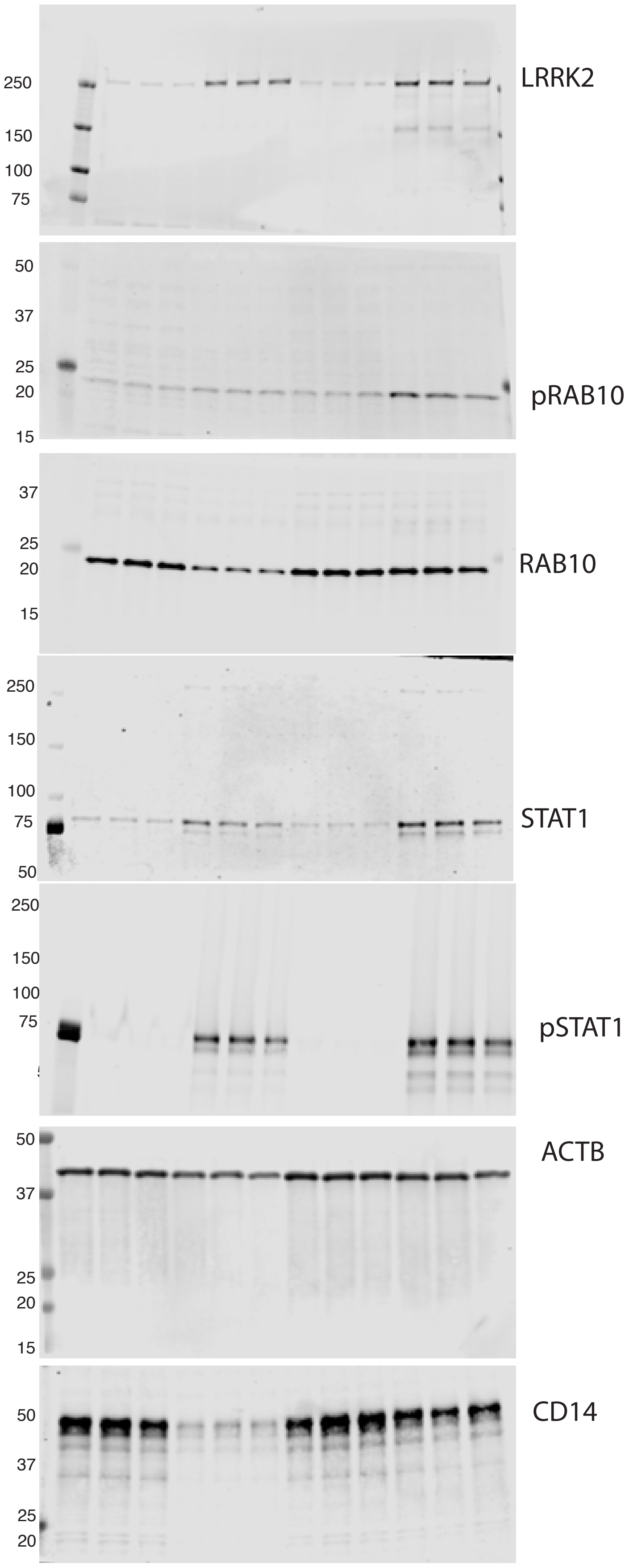

Figure 6c

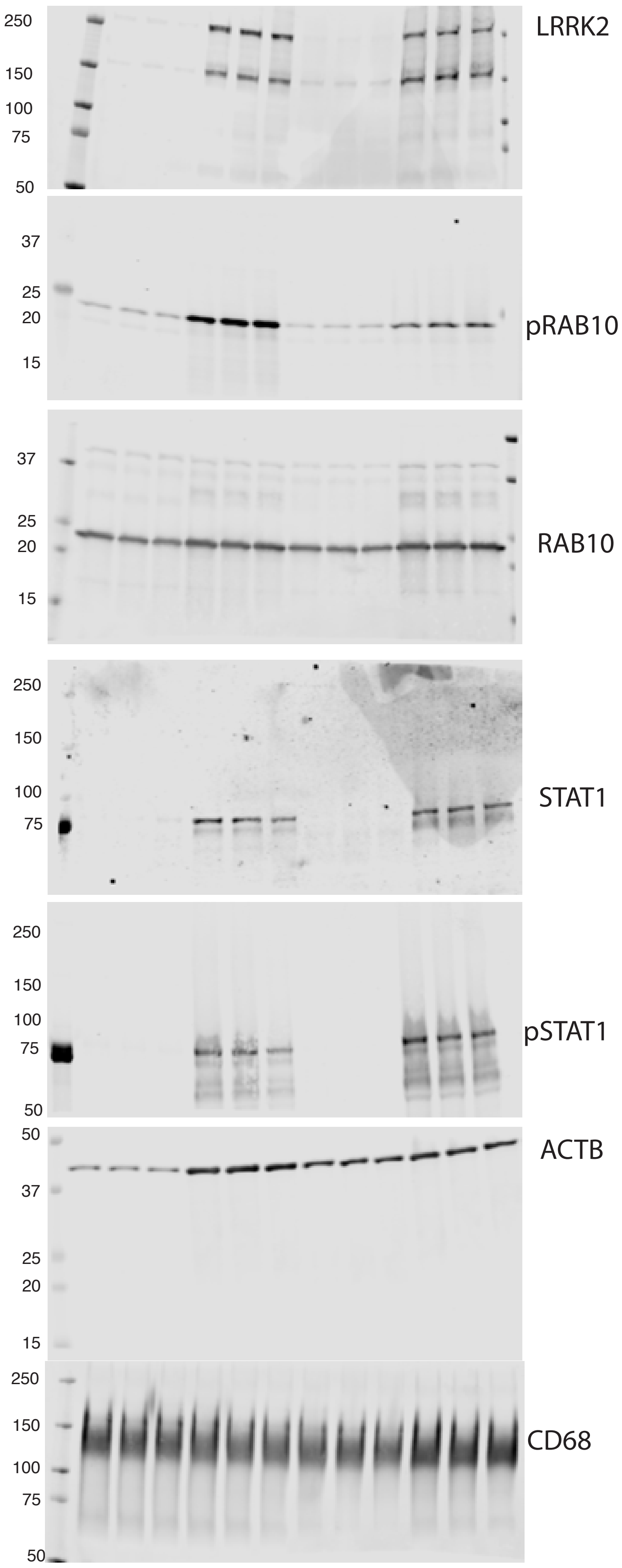

Figure 6b,c

Figure 6d

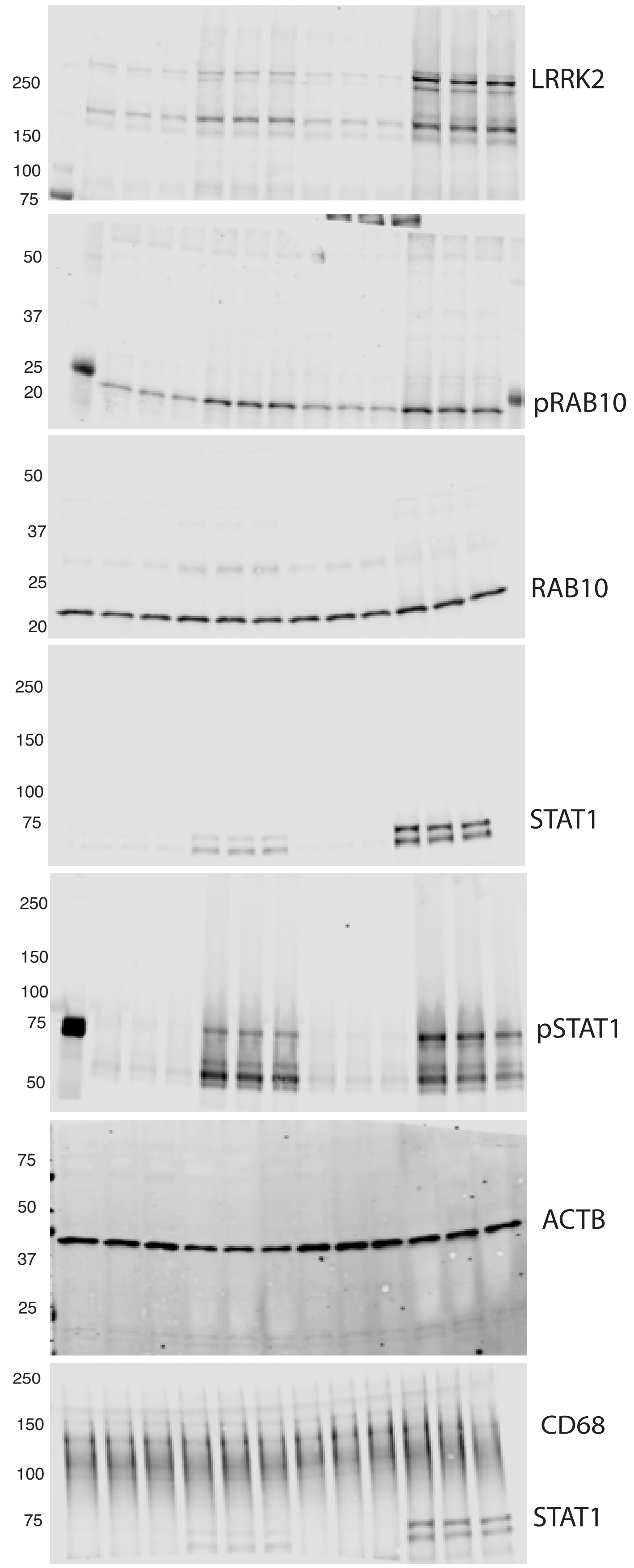

Figure 6k

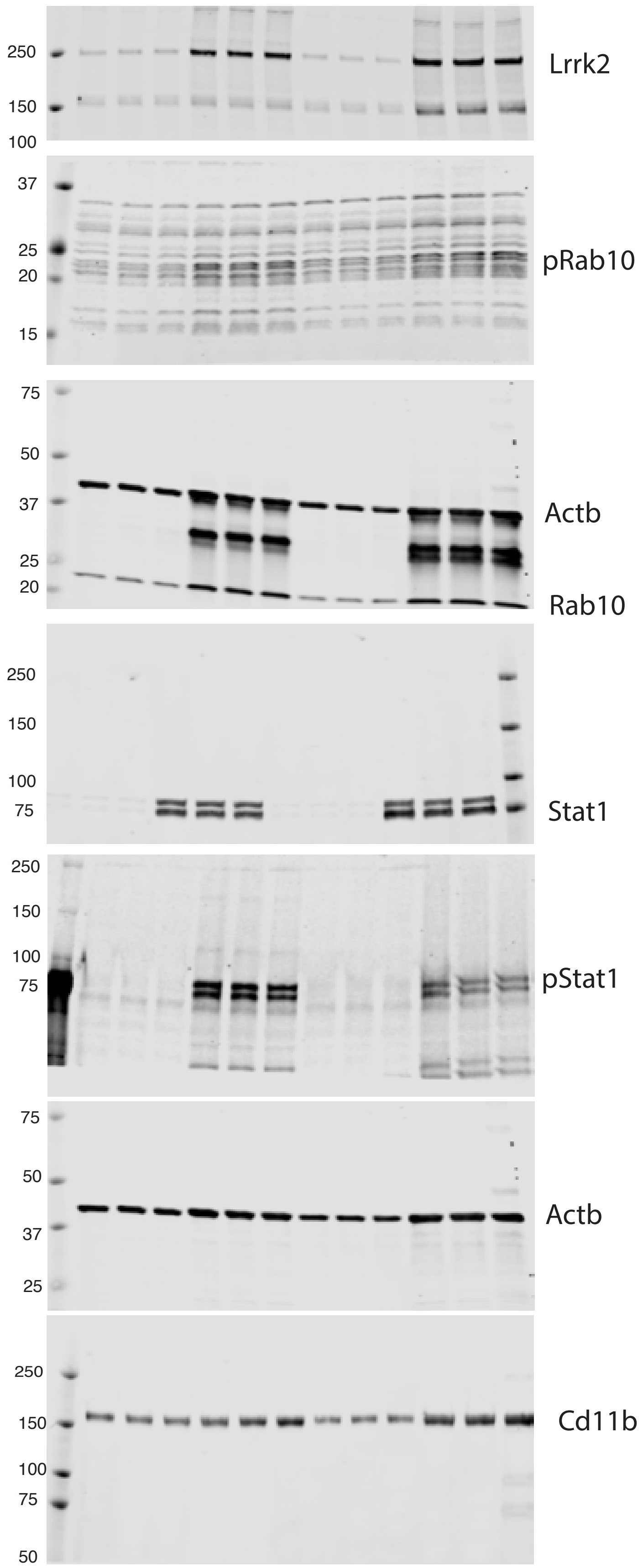

Figure 6d,k

Figure 6l

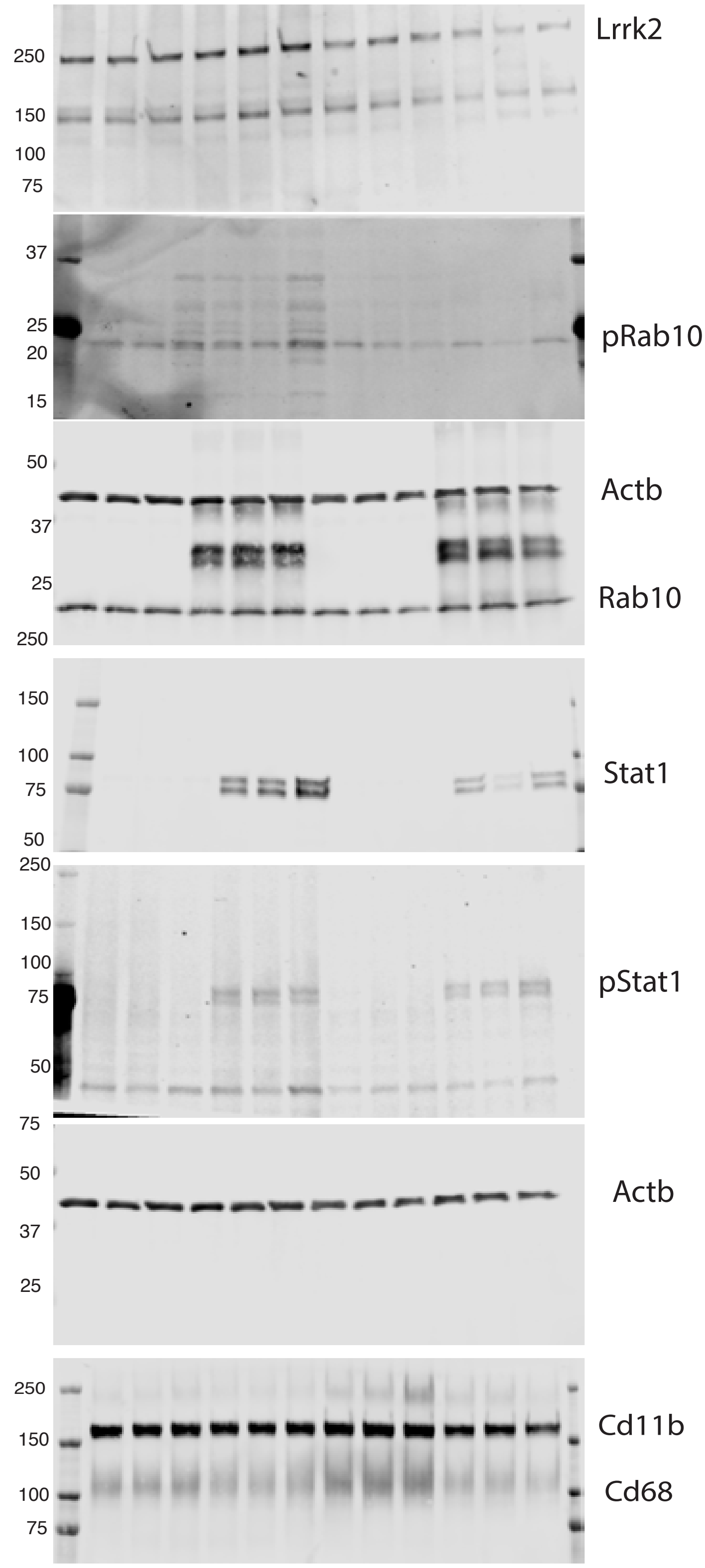

Figure 6m

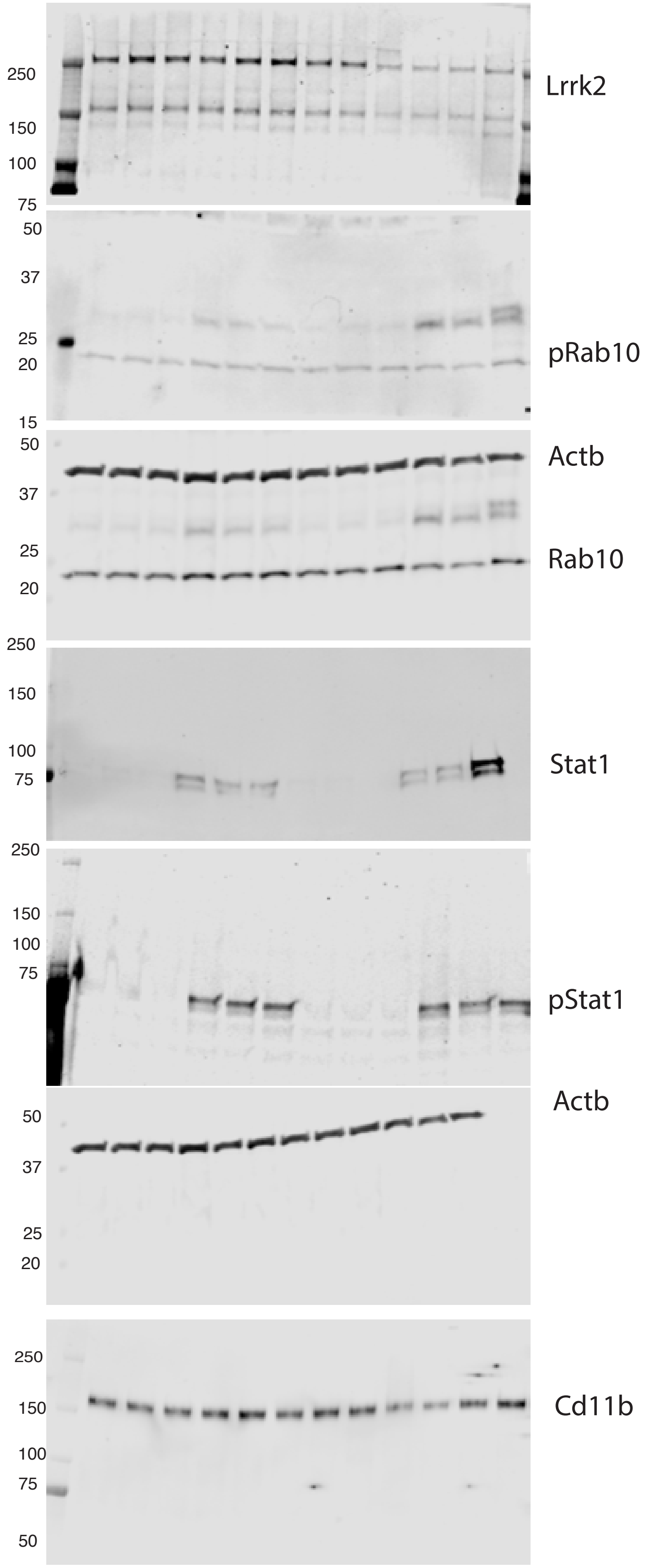

Figure 6l,m

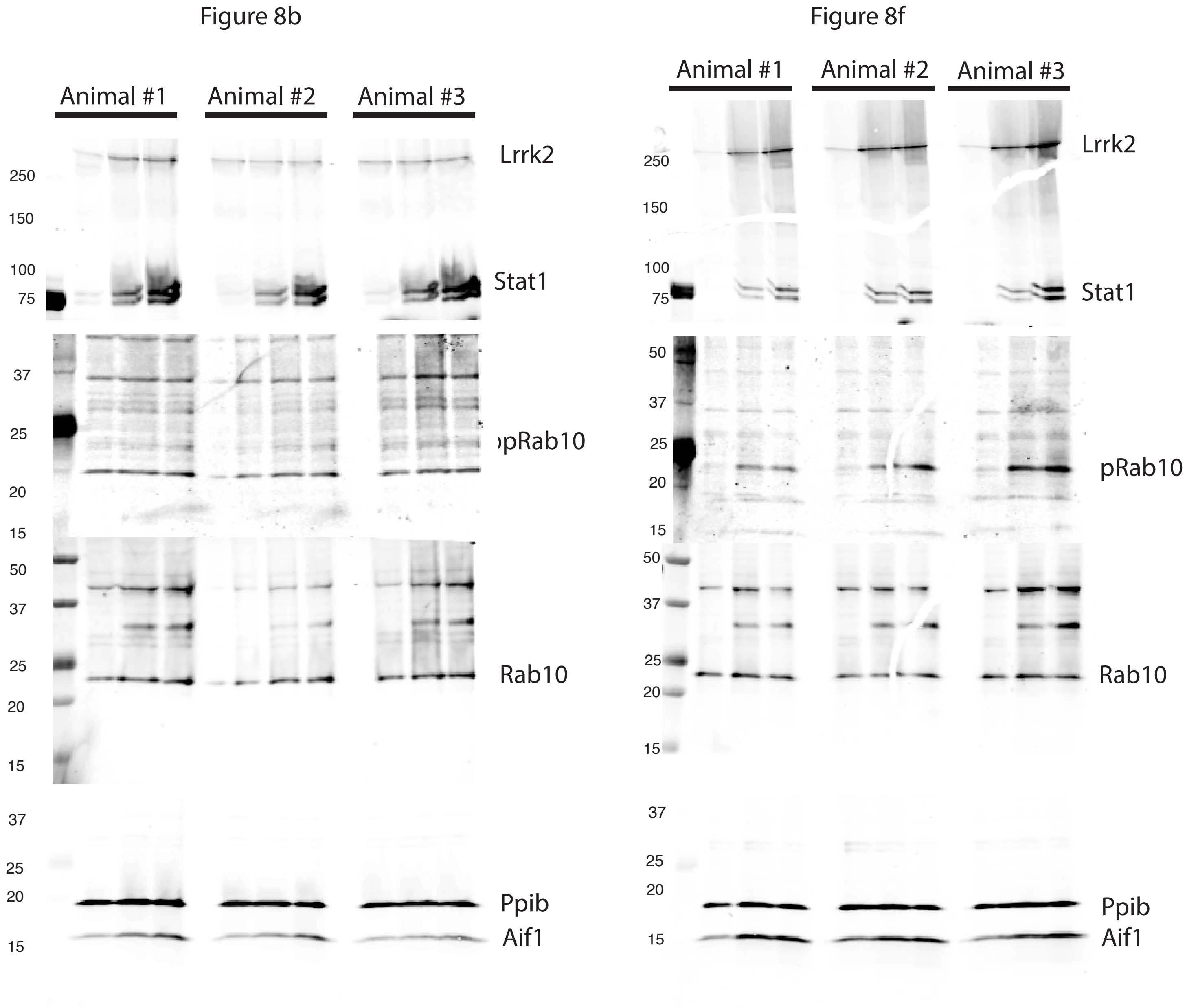

Figure 8b: Was generated from primary mouse microglia isolated from a wild-type animal #2, and was chosen for Figure 8b as a representative example.

Figure 8f: Was generated from primary mouse microglia isolated from a Lrrk2 knockout/transgenic for human LRRK2 (BAC) animal #1, and was chosen for Figure 8f as a representative example.

Figure S1a

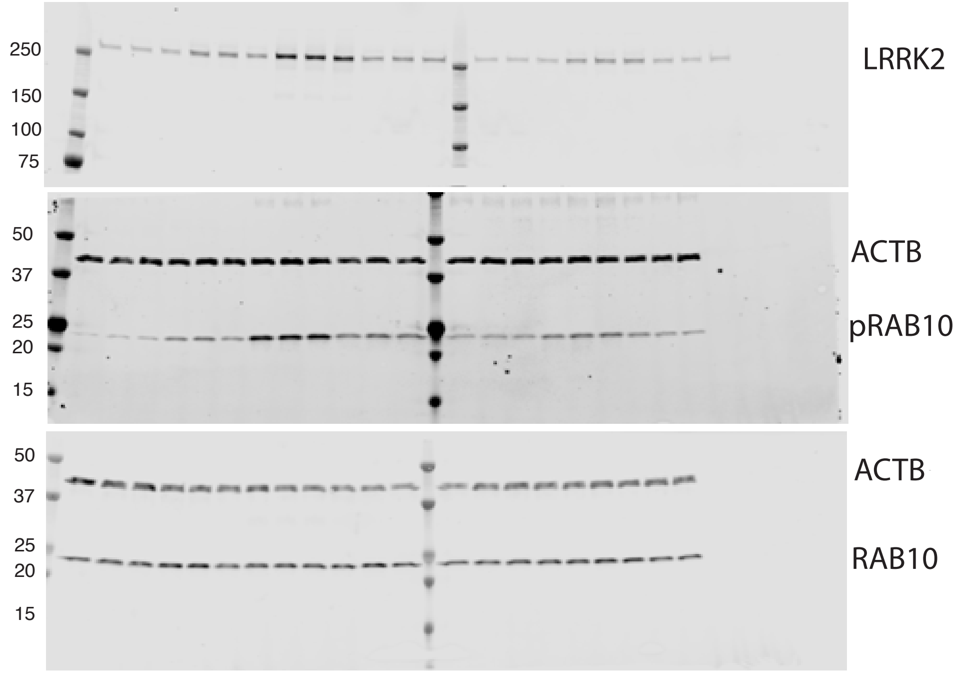

Figure S1d

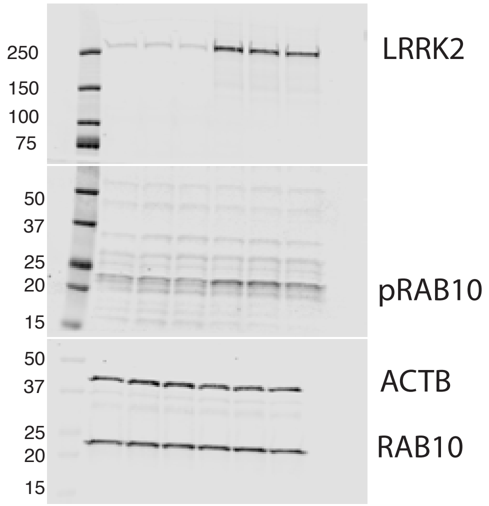

Figure S1g

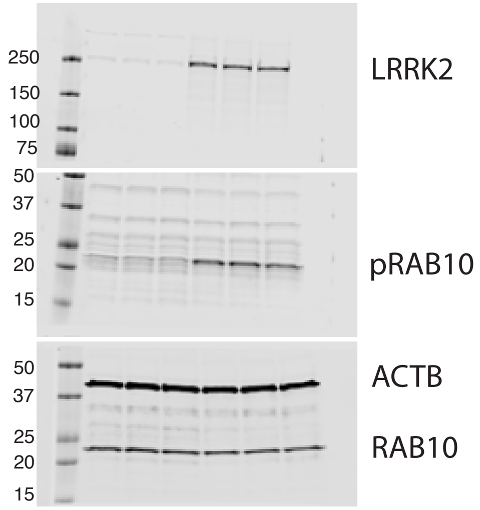

Figure S1j

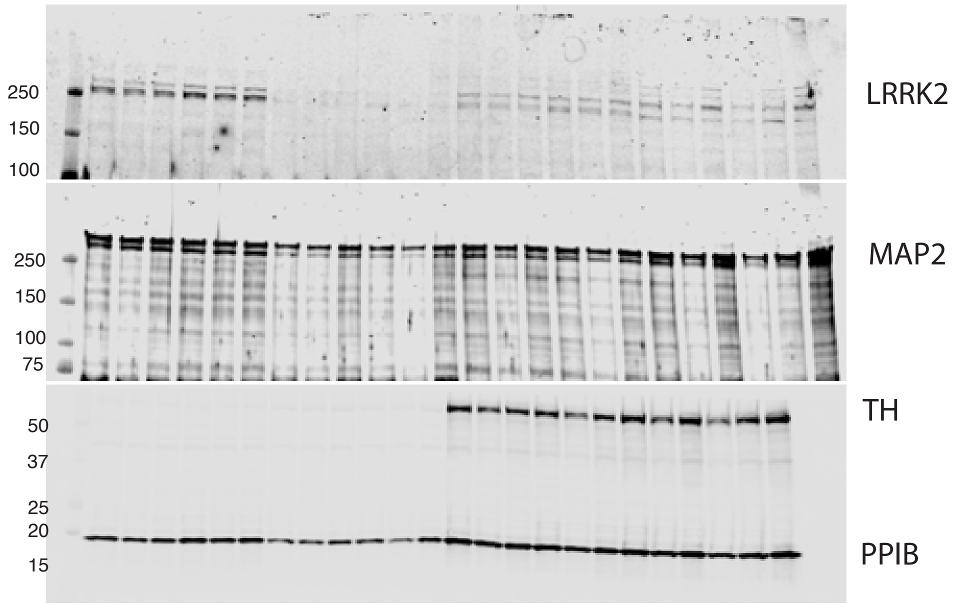

Figure S1a,d,g,j

Figure S4a

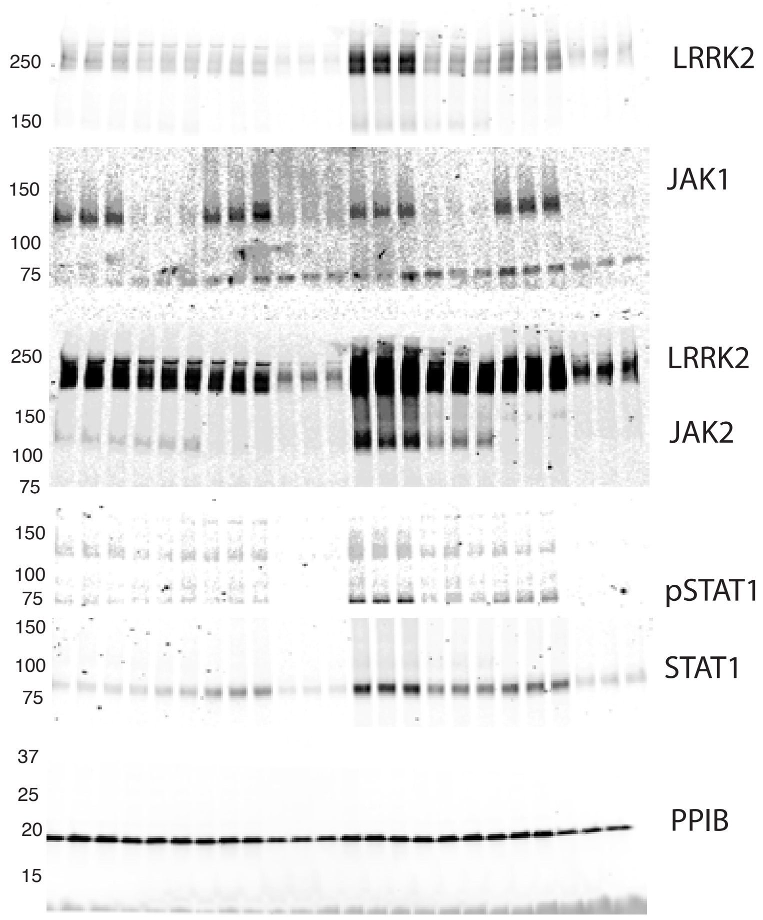

Figure S4f

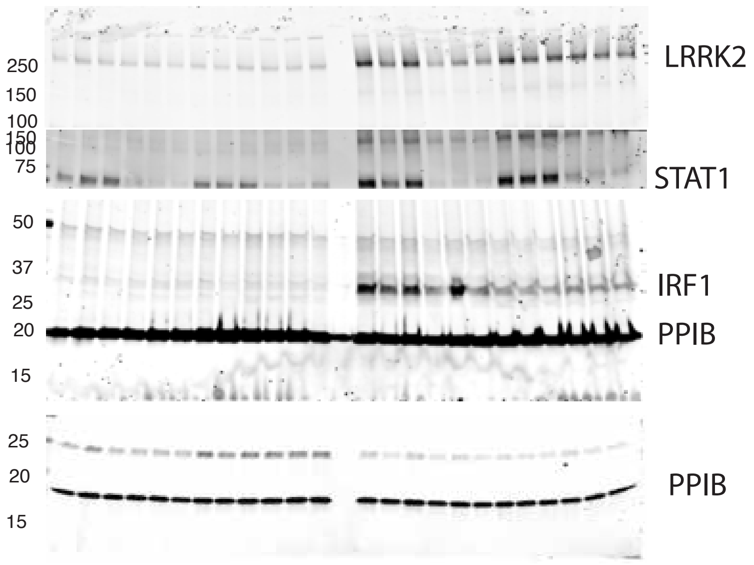

Figure S4a, f

Figure S7a

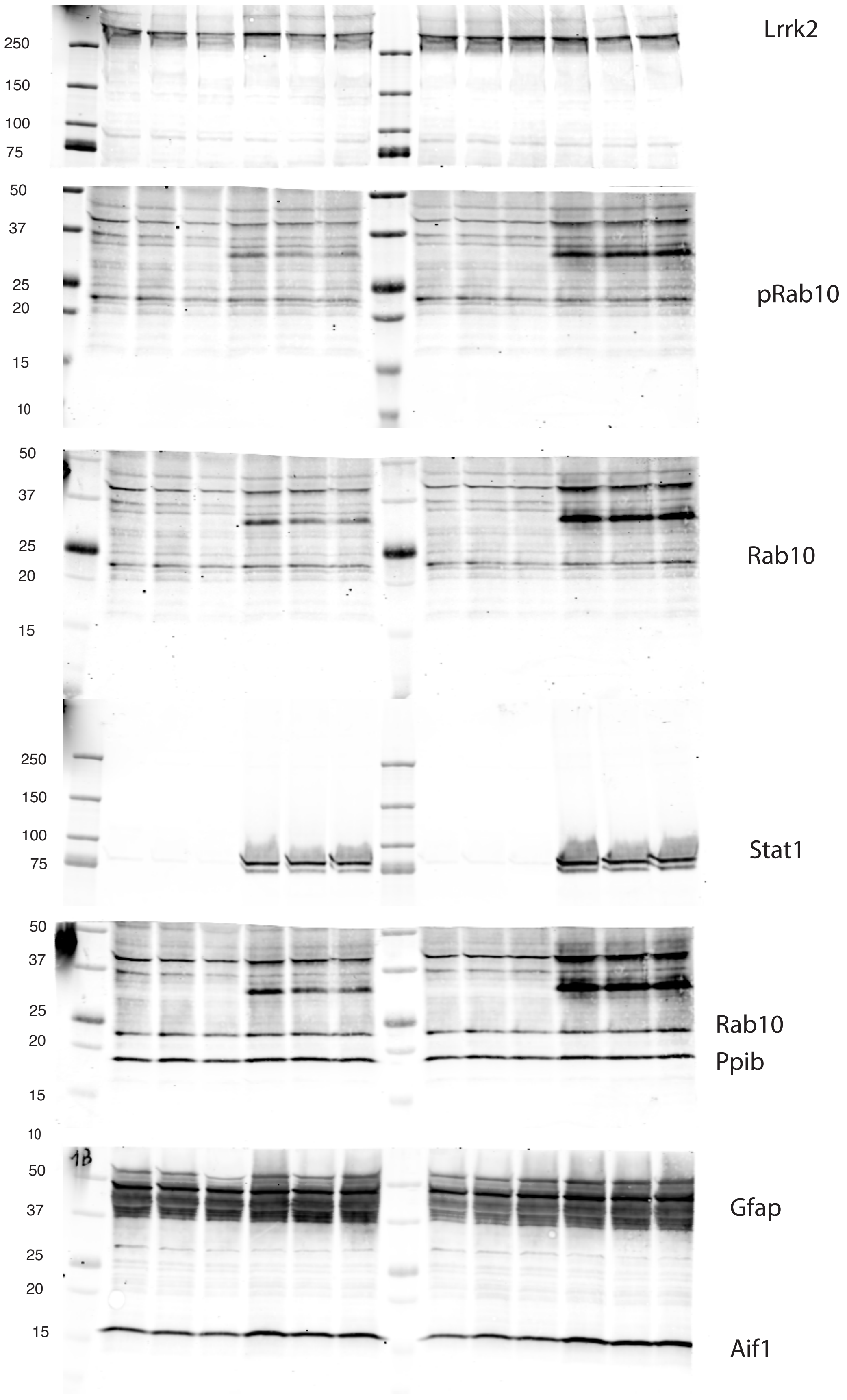

Figure S7a

Figure S7d

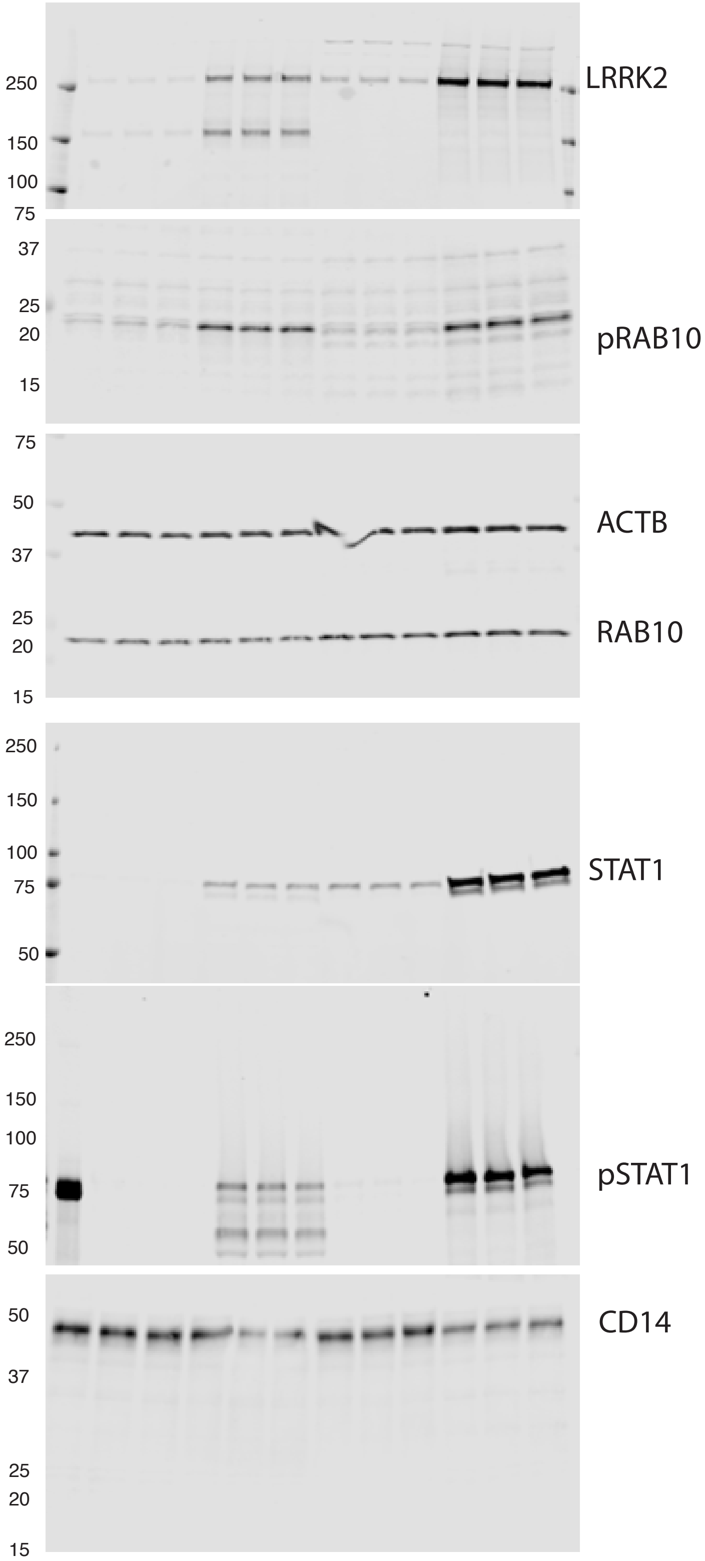

Figure S7e

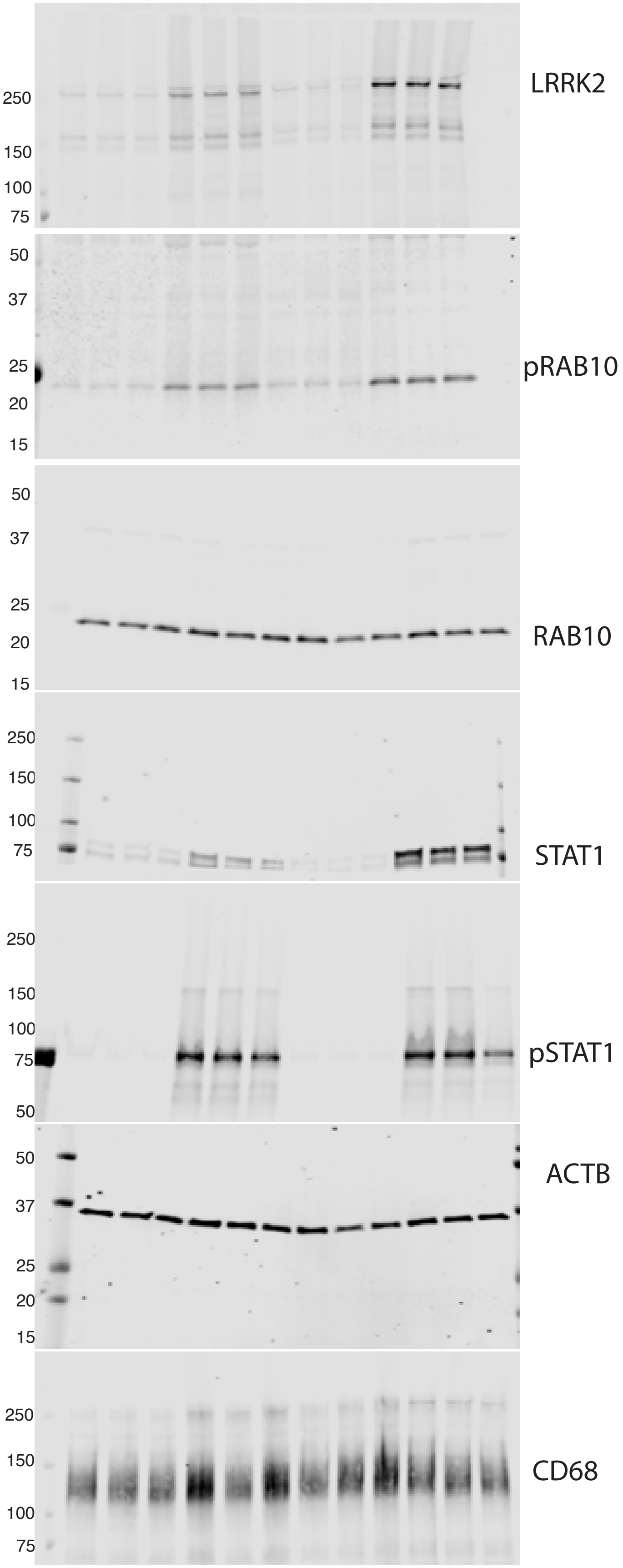

Figure S7d,e

Figure S7f

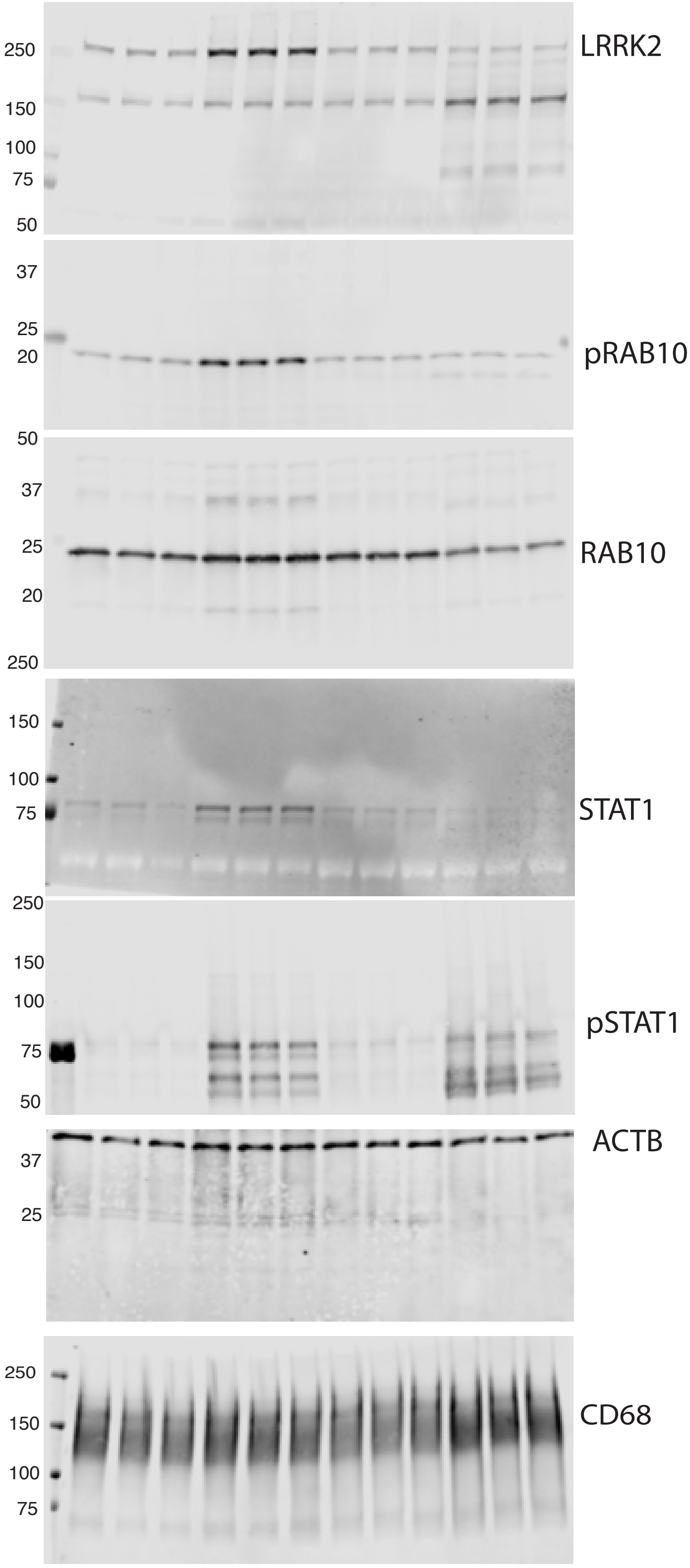

Figure S7m

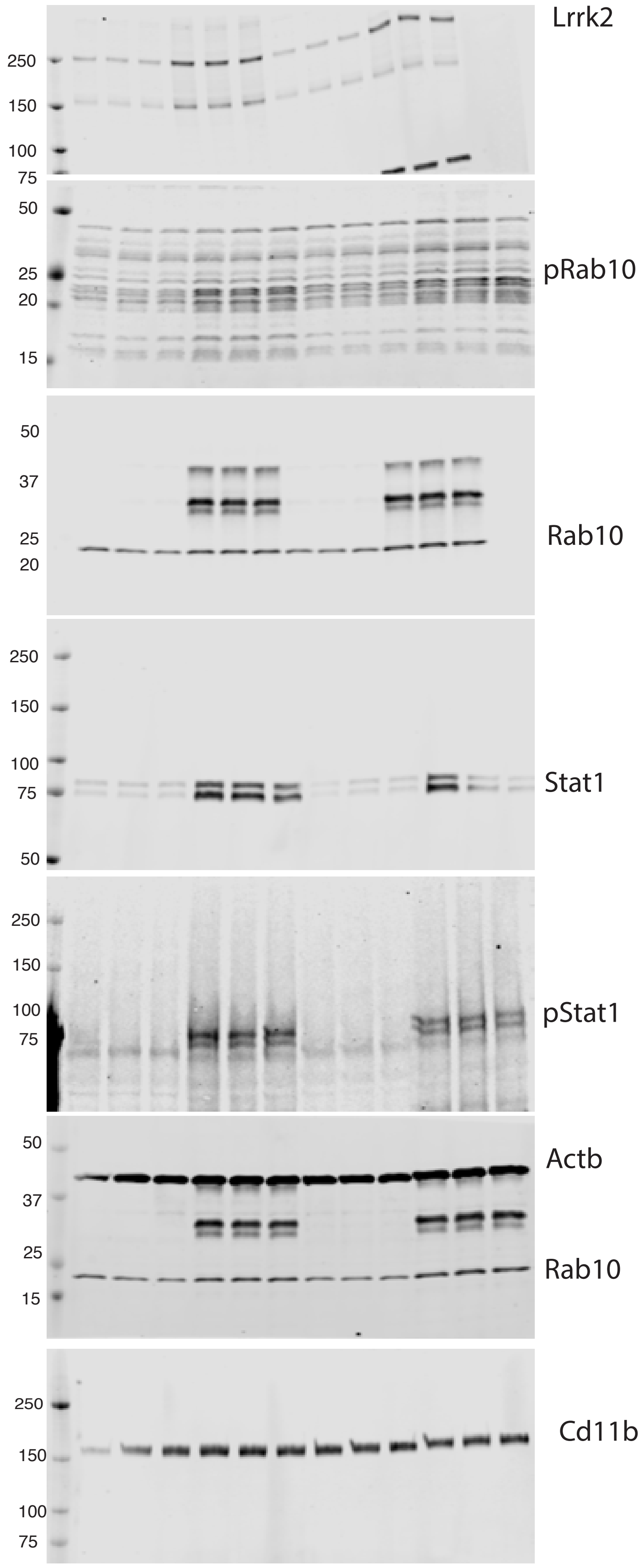

Figure S7f,m

Figure S7n

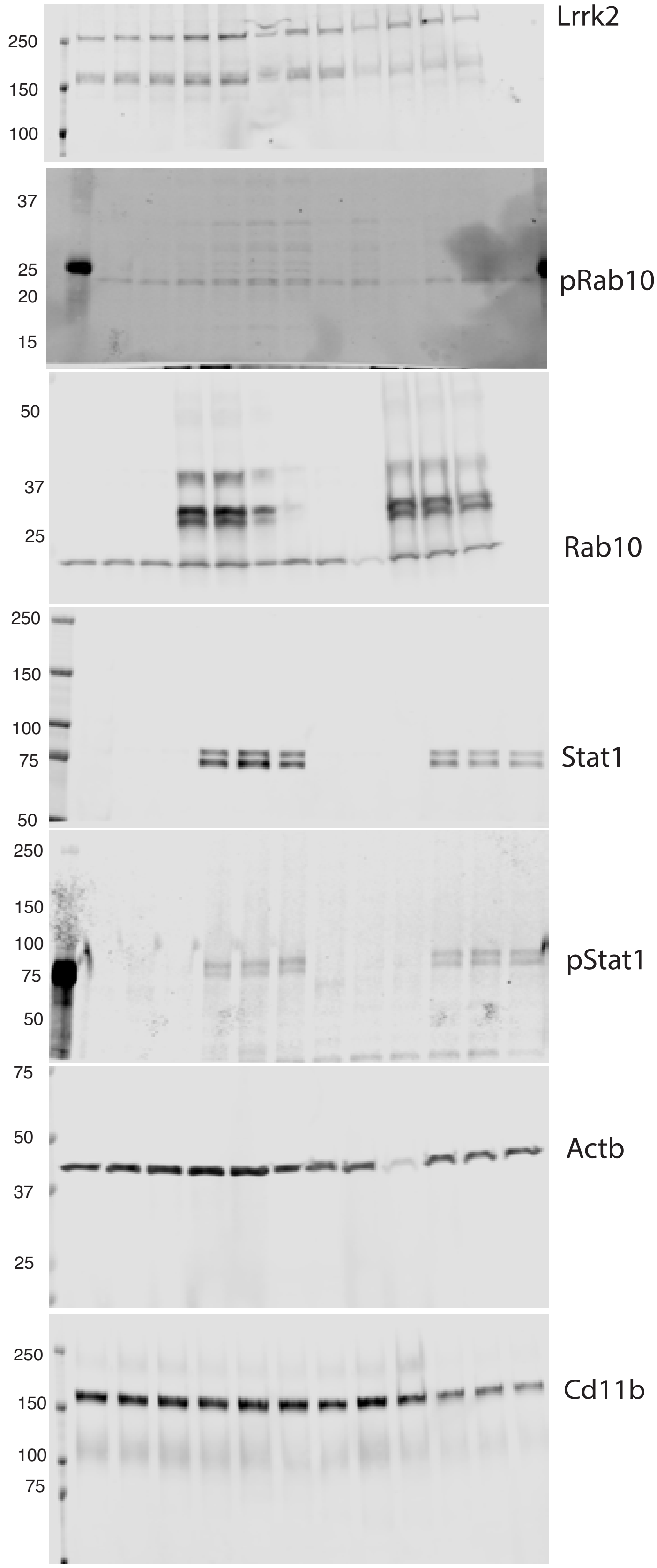

Figure S7o

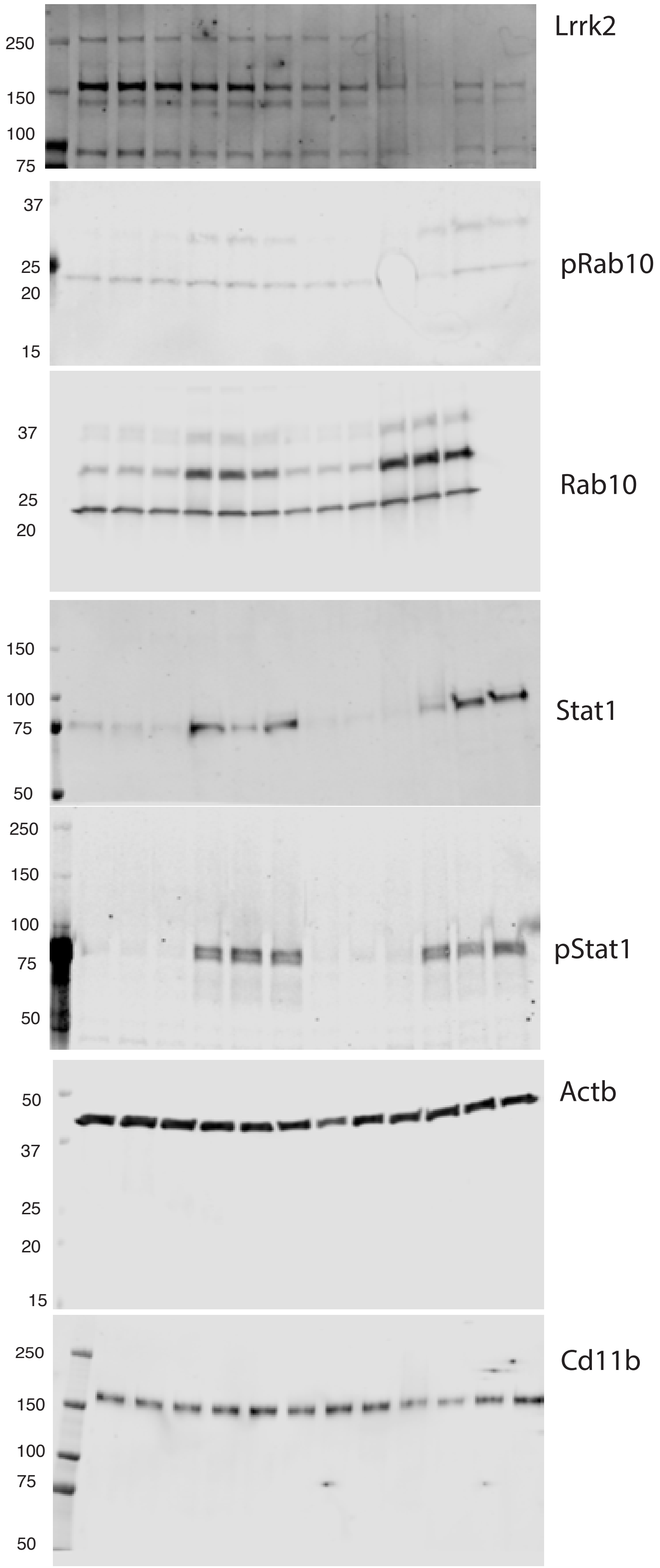

Figure S7n,0

Figure S9a

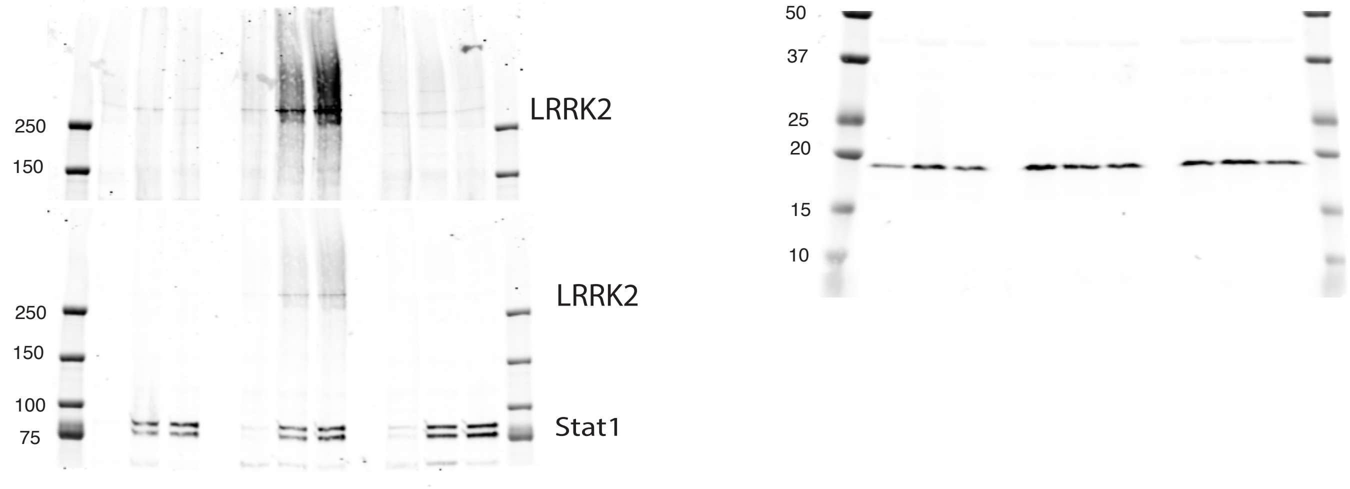

Figure S9d

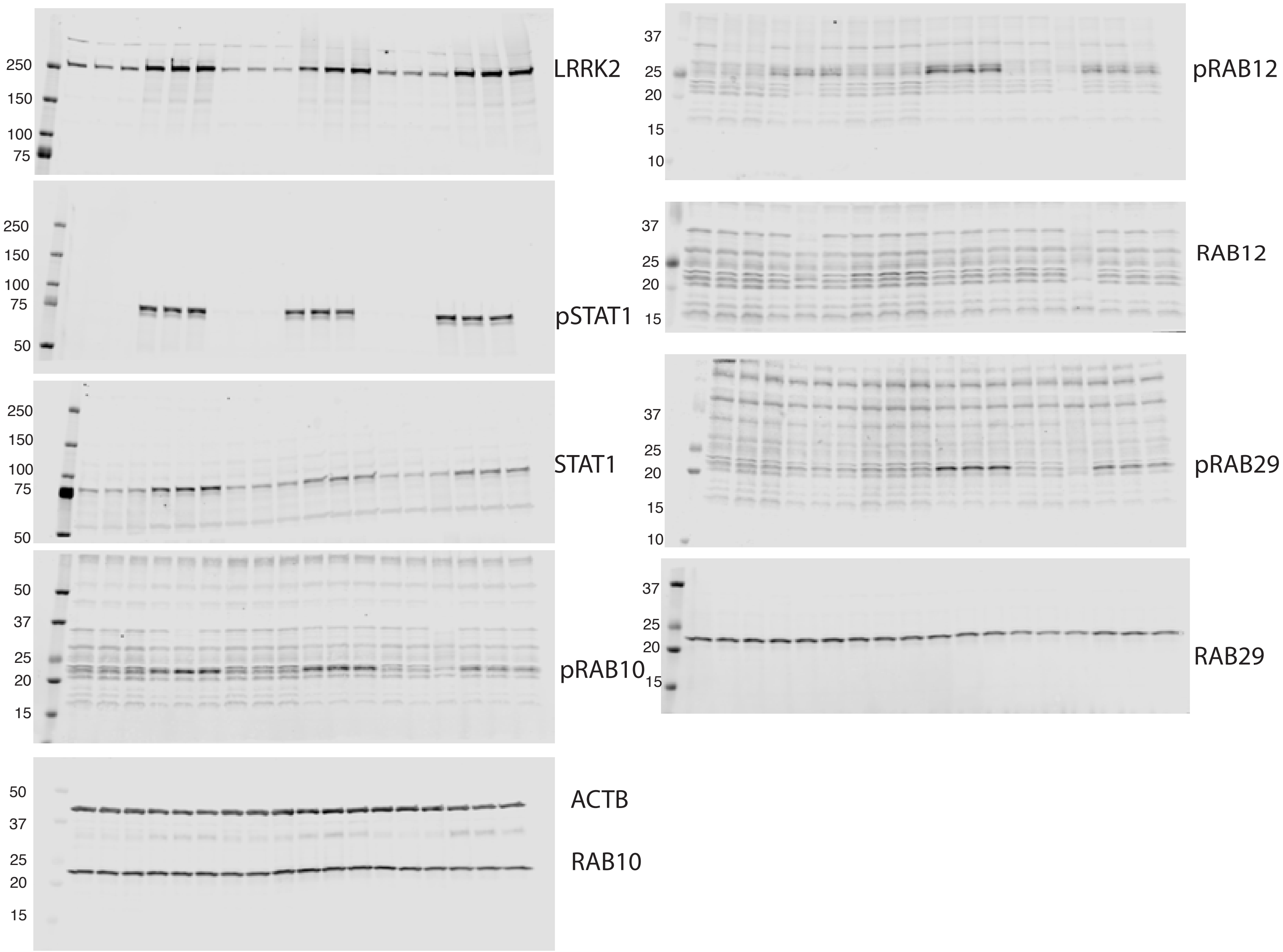

Figure S9
