# Supplementary figures and images for: Inflammatory signaling differentially changes chromatin accessibility and gene expression of the PD- associated kinase LRRK2 between human and mice
Source: Mol Neurodegener. 2026 Mar 18;21:24. doi: 10.1186/s13024-026-00938-0 (PMC13112740; doi:10.1186/s13024-026-00938-0)

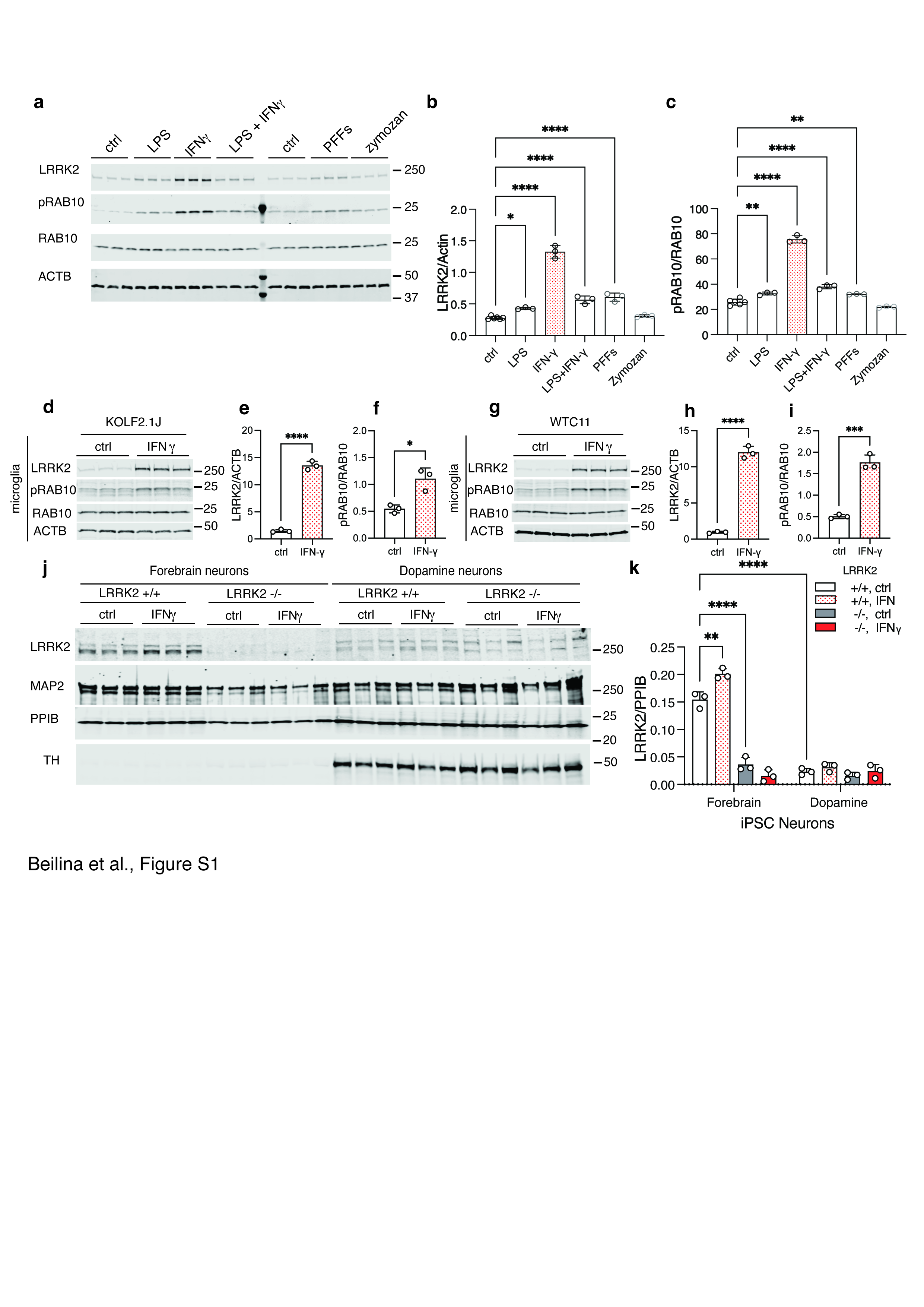

Supplement: Supplementary file 1 — Supplementary Material 1 [file 13024_2026_938_MOESM1_ESM.zip › Supplementary/Figure S1 R1.tif]

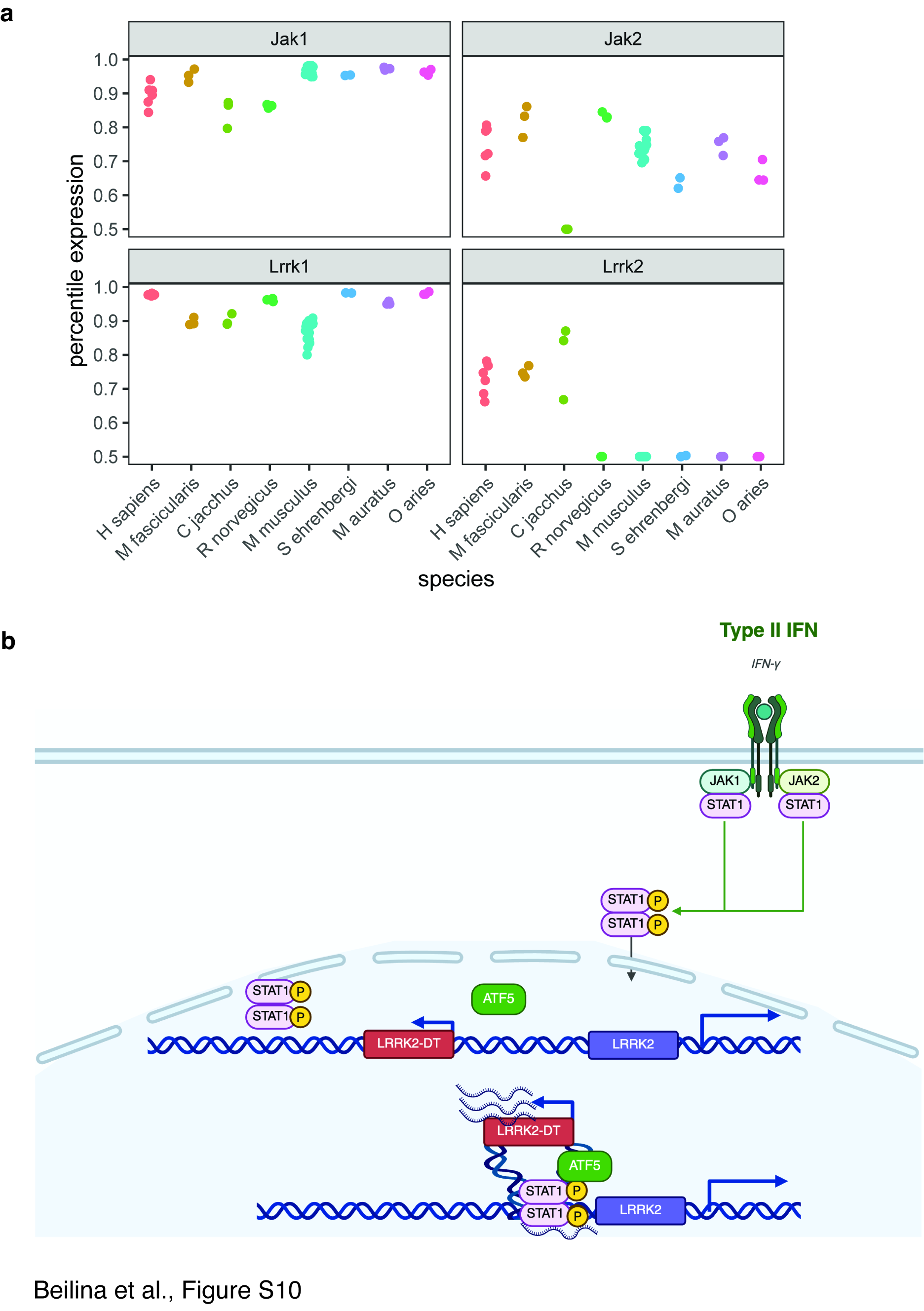

Supplement: Supplementary file 1 — Supplementary Material 1 [file 13024_2026_938_MOESM1_ESM.zip › Supplementary/Figure S10 R1.tif]

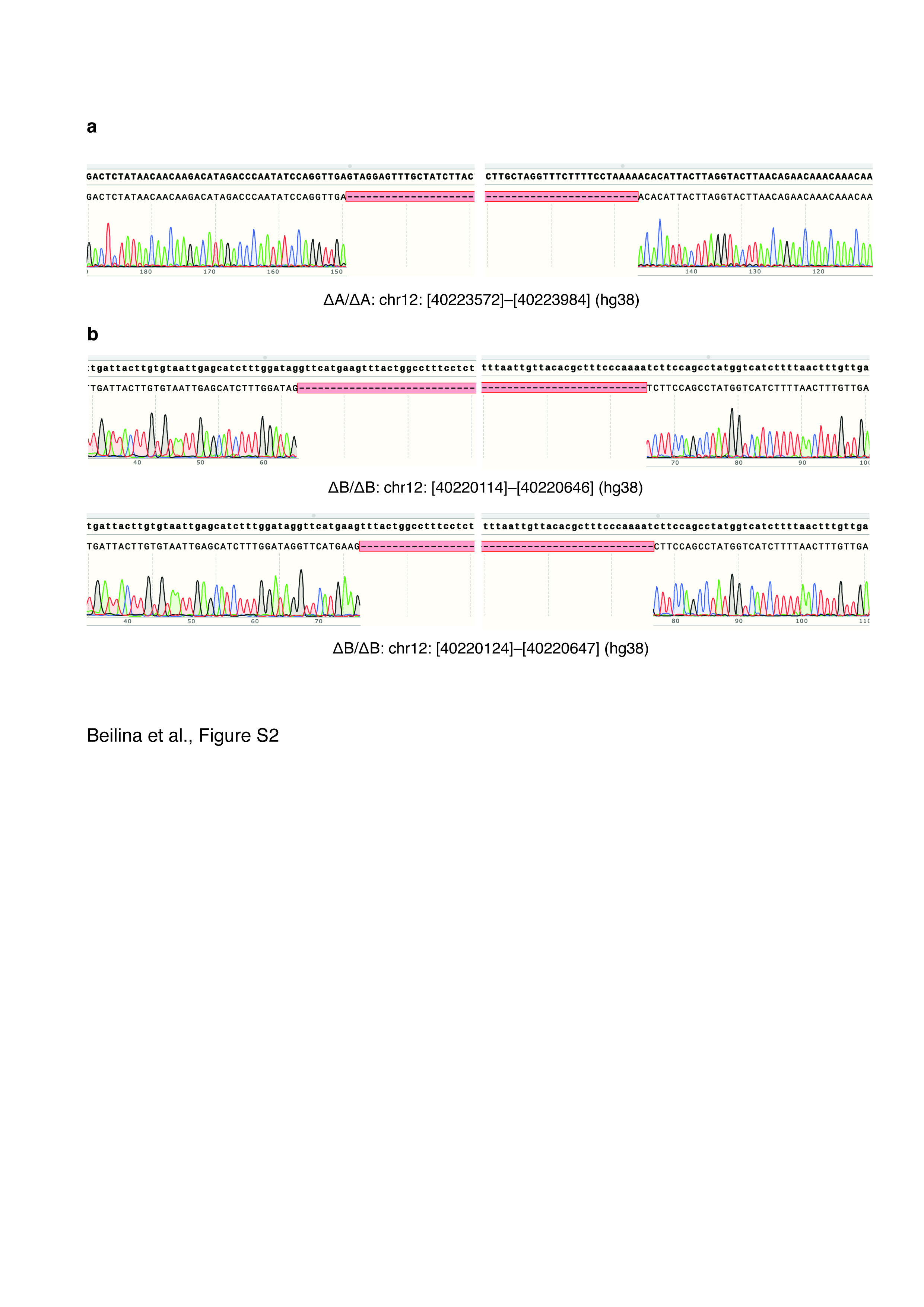

Supplement: Supplementary file 1 — Supplementary Material 1 [file 13024_2026_938_MOESM1_ESM.zip › Supplementary/Figure S2 R1.tif]

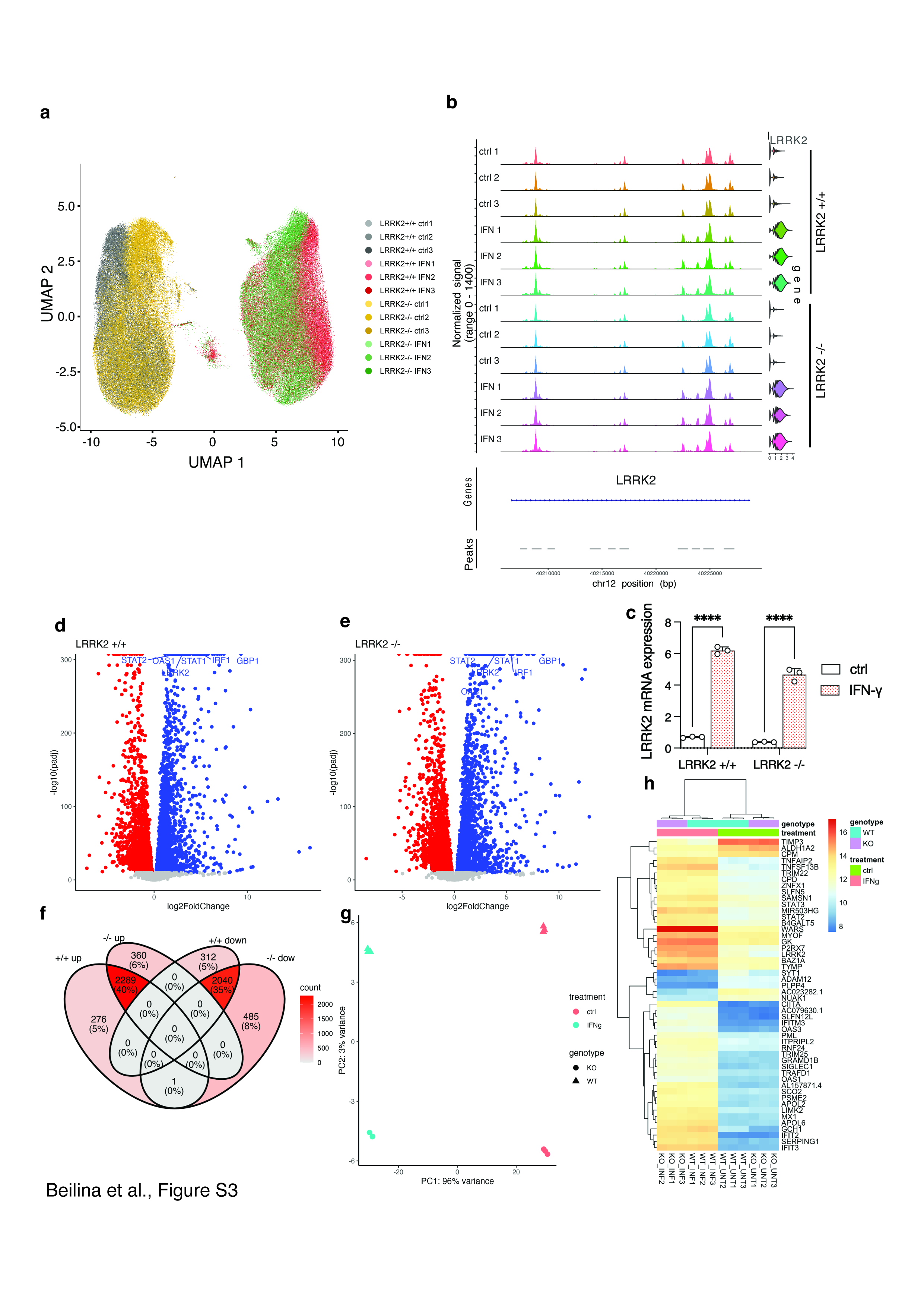

Supplement: Supplementary file 1 — Supplementary Material 1 [file 13024_2026_938_MOESM1_ESM.zip › Supplementary/Figure S3 R1.tif]

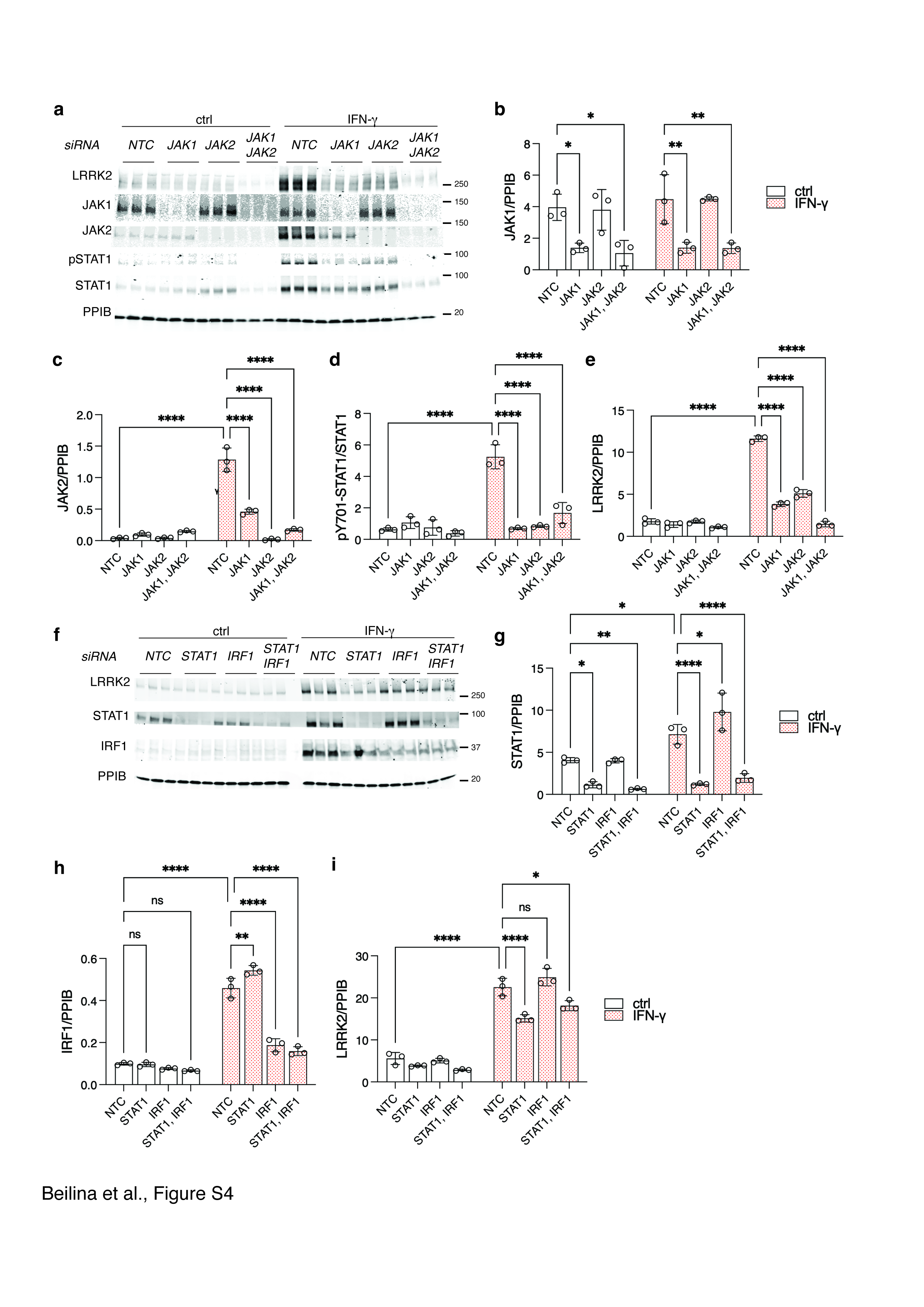

Supplement: Supplementary file 1 — Supplementary Material 1 [file 13024_2026_938_MOESM1_ESM.zip › Supplementary/Figure S4 R1.tif]

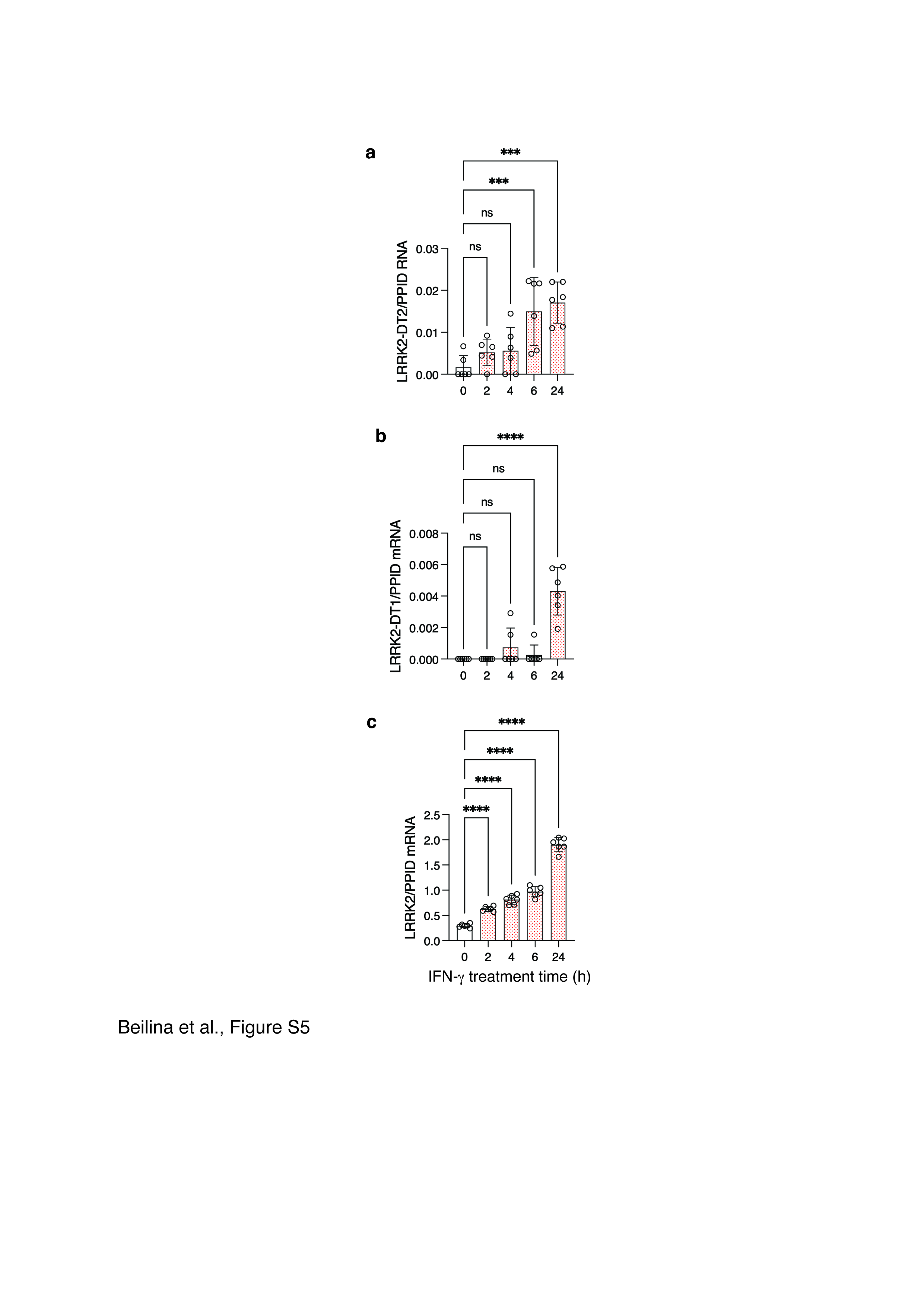

Supplement: Supplementary file 1 — Supplementary Material 1 [file 13024_2026_938_MOESM1_ESM.zip › Supplementary/Figure S5 R1.tif]

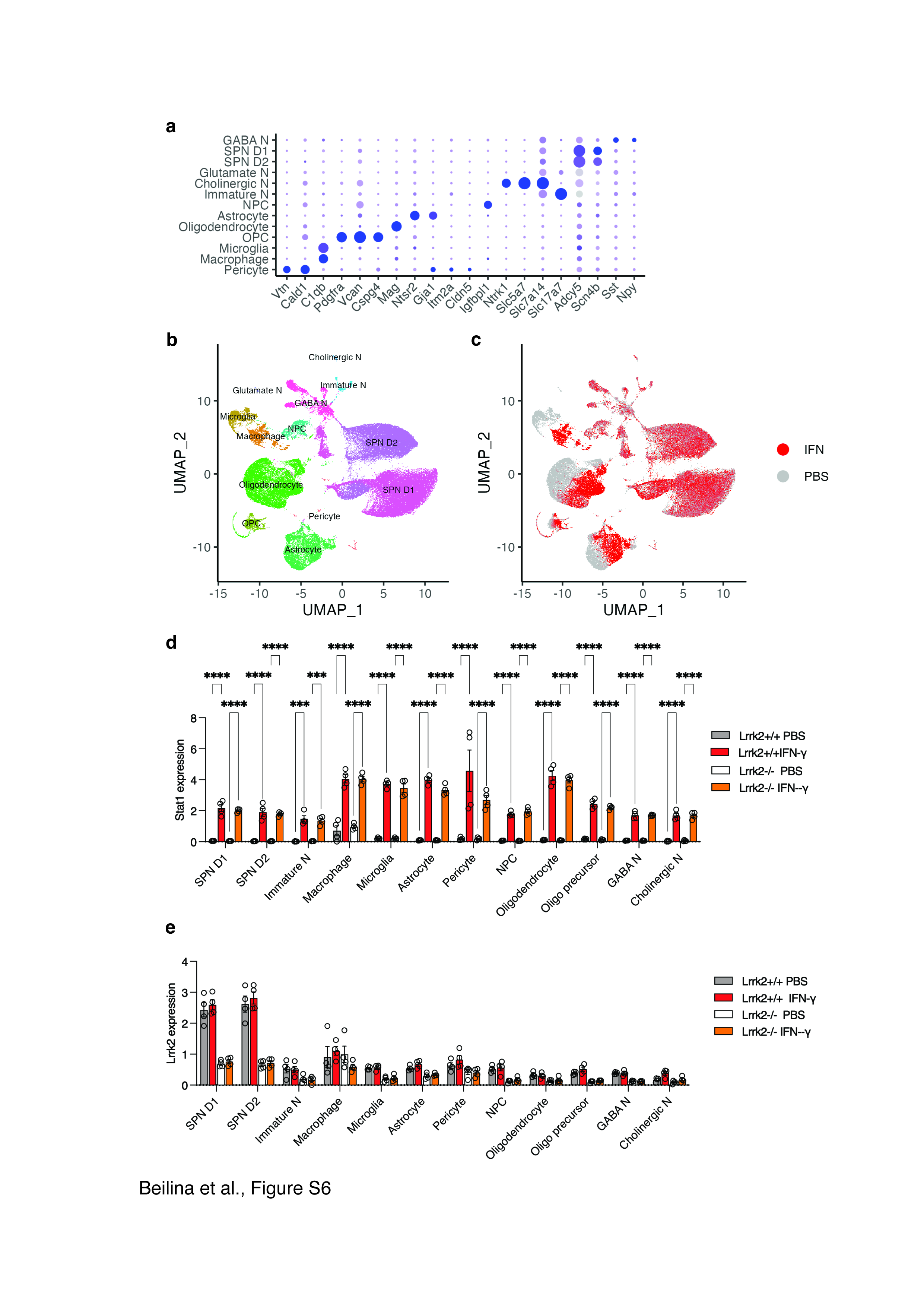

Supplement: Supplementary file 1 — Supplementary Material 1 [file 13024_2026_938_MOESM1_ESM.zip › Supplementary/Figure S6 R1.tif]

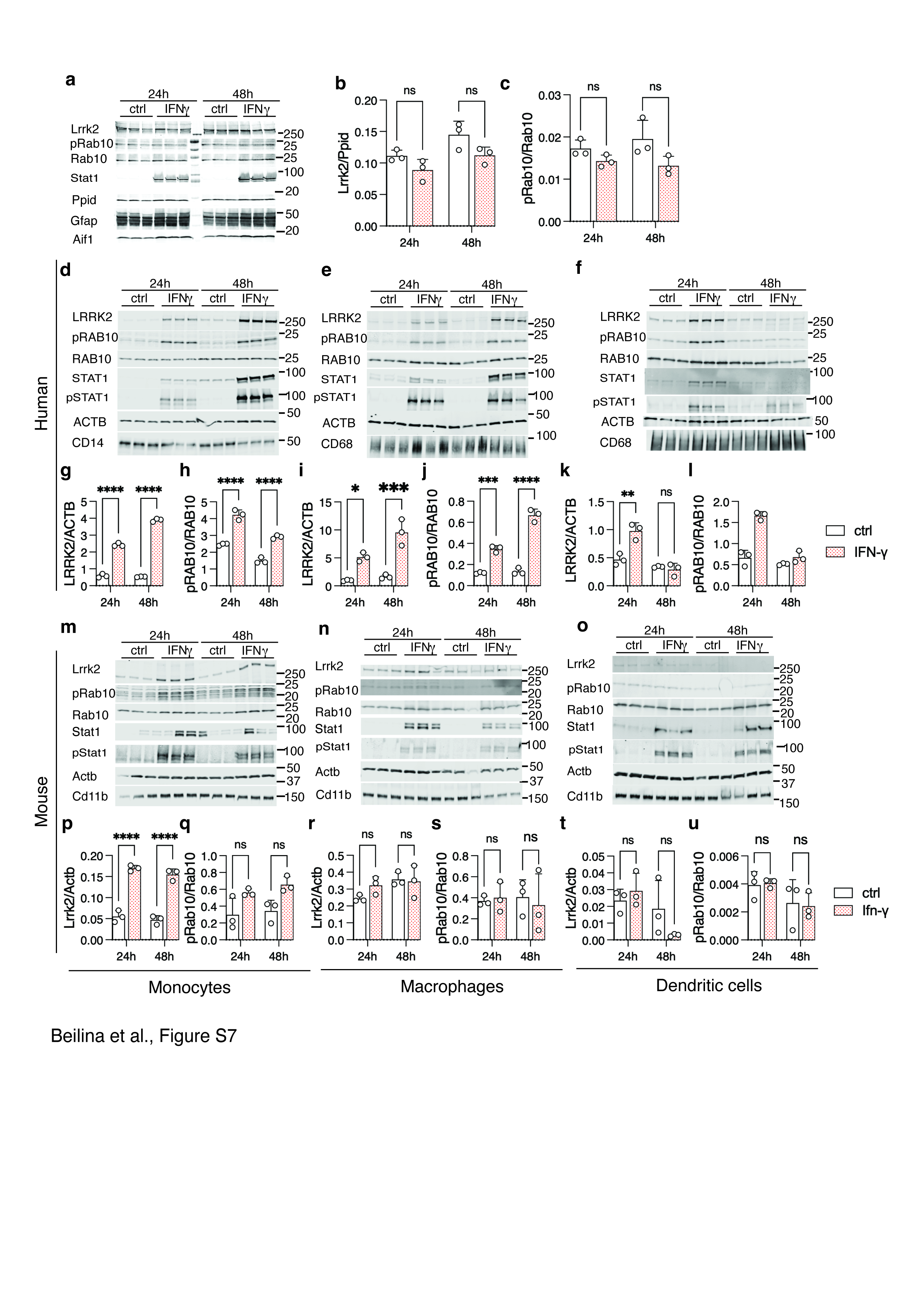

Supplement: Supplementary file 1 — Supplementary Material 1 [file 13024_2026_938_MOESM1_ESM.zip › Supplementary/Figure S7 R1.tif]

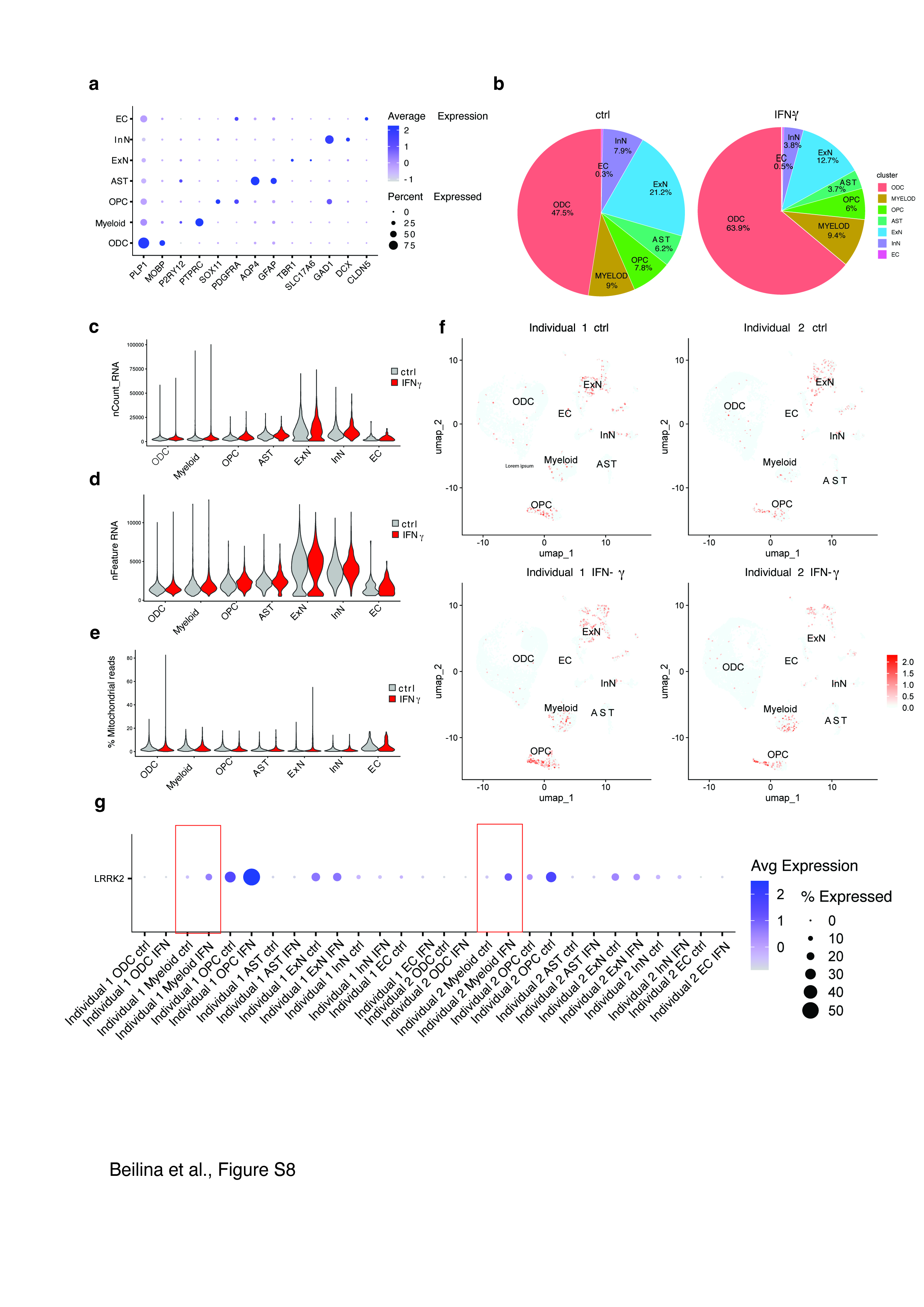

Supplement: Supplementary file 1 — Supplementary Material 1 [file 13024_2026_938_MOESM1_ESM.zip › Supplementary/Figure S8 R1.tif]

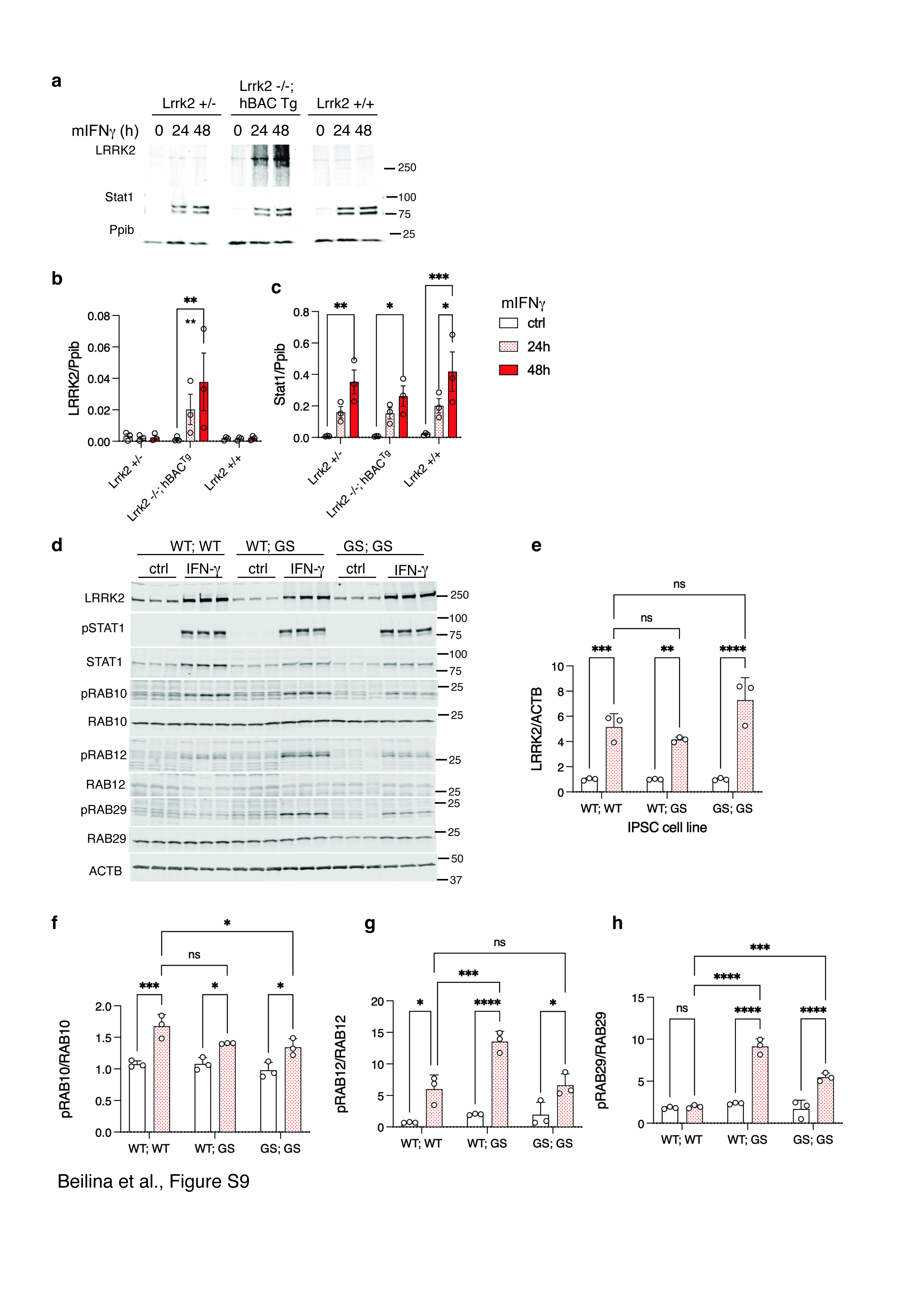

Supplement: Supplementary file 1 — Supplementary Material 1 [file 13024_2026_938_MOESM1_ESM.zip › Supplementary/Figure S9 R1.tif]
